# Cross-species single-cell transcriptomic analysis reveals pre-gastrulation developmental differences among pigs, monkeys, and humans

**DOI:** 10.1038/s41421-020-00238-x

**Published:** 2021-02-02

**Authors:** Tianbin Liu, Jie Li, Leqian Yu, Hai-Xi Sun, Jing Li, Guoyi Dong, Yingying Hu, Yong Li, Yue Shen, Jun Wu, Ying Gu

**Affiliations:** 1 BGI Education Center, University of Chinese Academy of Sciences, Shenzhen, Guangdong 518083 China; 2 BGI-Shenzhen, Shenzhen, Guangdong 518083 China; 3 Guangdong Provincial Key Laboratory of Genome Read and Write, BGI-Shenzhen, Shenzhen, Guangdong 518120 China; 4 Department of Molecular Biology, University of Texas Southwestern Medical Center, Dallas, TX 75390 USA; 5 Hamon Center for Regenerative Science and Medicine, University of Texas Southwestern Medical Center, Dallas, TX 75390 USA; 6 BGI Institute of Applied Agriculture, BGI-Shenzhen, Shenzhen, Guangdong 518120 China; 7 Institute for Stem cell and Regeneration, Chinese Academy of Sciences, Beijing, 100101 China

**Keywords:** Pluripotency, Cell growth, Cell signalling

## Abstract

Interspecies blastocyst complementation enables organ-specific enrichment of xenogeneic pluripotent stem cell (PSC) derivatives, which raises an intriguing possibility to generate functional human tissues/organs in an animal host. However, differences in embryo development between human and host species may constitute the barrier for efficient chimera formation. Here, to understand these differences we constructed a complete single-cell landscape of early embryonic development of pig, which is considered one of the best host species for human organ generation, and systematically compared its epiblast development with that of human and monkey. Our results identified a developmental coordinate of pluripotency spectrum among pigs, humans and monkeys, and revealed species-specific differences in: (1) pluripotency progression; (2) metabolic transition; (3) epigenetic and transcriptional regulations of pluripotency; (4) cell surface proteins; and (5) trophectoderm development. These differences may prevent proper recognition and communication between donor human cells and host pig embryos, resulting in low integration and survival of human cells. These results offer new insights into evolutionary conserved and divergent processes during mammalian development and may be helpful for developing effective strategies to overcome low human–pig chimerism, thereby enabling the generation of functional human organs in pigs in the future.

## Introduction

Shortage of human organs for transplantation represents one of the largest unmet medical needs worldwide, which is expected to increase. Human pluripotent stem cells (PSCs) derived from blastocysts^1^ or generated via somatic reprograming^2^ offer a potential unlimited cellular source to generate donor organs. Despite years of research, it remains improbable to generate fully functional organs in vitro. To bypass this obstacle, an in vivo approach known as interspecies blastocyst complementation has been developed, which involves the injection of PSCs from one species into an organogenesis-disabled blastocyst of another species. During embryogenesis, the mutant host embryo provides an emptied “developmental organ niche” for the donor PSCs to occupy, and thereby generating a chimeric organ enriched with cells from the donor species^3,4^. Interspecies blastocyst complementation raises an intriguing possibility to generate functional human tissues and organs in an animal host. One good candidate host animal is the pigs due to their resemblance to humans in anatomy, physiology, organ size and cell cycle characteristics^5^.

The key to success for interspecies blastocyst complementation is the chimera competency of donor PSCs in the host species. Rat and mouse PSCs can efficiently contribute to chimera formation in mouse and rat, respectively, thus leading to the successful generation of rat pancreas, thymus, eye and fetal heart and endothelial tissues in mice^6^, and mouse pancreas and kidney in rats via interspecies blastocyst complementation^7–11^. With regard to human and pig blastocyst complementation, however, accumulating evidence have shown that human PSCs inefficiently contributed to chimera formation in early pig embryos (E21–E28) and the level of chimerism was far lower than that between rat and mouse, regardless of pluripotent states, injection timing and number of cells injected^10^. These results indicate a major xenogeneic barrier exists between human and pig during early embryogenesis.

Pigs are considered more closer to humans than rodents in pre-gastrulation development: (1) they both have a protracted developmental period; (2) epiblast cells in both humans and pigs self-organize into a flat bilaminar disc rather than a cup-shaped epithelium; (3) they share a number of regulatory mechanisms underpinning early lineage segregation, pluripotency regulation, primordial germ cell specification, and X-inactivation^12,13^. Notwithstanding these similarities, notable species-specific differences have been recognized: (1) in contrast to hemochorial placenta found in humans, pigs have an epitheliochorial placenta; (2) unlike human, early pig development is characterized by dramatic and rapid elongation of the developing conceptus within the uterine lumen between 8–12 days of gestation. During this period, pig conceptus grows from an spherical shape with 2–6 mm in diameter on day 10 to tubular and then elongated filamentous forms approximately 100–150 mm in length on day 12^14,15^. These and other differences may limit the degree of donor human PSCs to survive, proliferate and differentiate inside a developing pig embryo.

Single-cell RNA sequencing (scRNA-seq) technology has been used to chart the transcriptome landscapes and provided insights into the molecular regulation of early mammalian development^12,16–19^. Cross-species transcriptome comparison of early embryonic tissues at single-cell level also helped uncover molecular basis for many conserved and divergent developmental processes among several mammalian species^18^.

Here, we performed scRNA-seq analysis of pig embryos isolated from 4 different stages of pre-gastrulation development, which include early blastocysts (E5–6), late blastocyst (E7–8), spherical (E10–11) and filamentous stages (E12–13). We also performed cross-species transcriptome comparison between pigs, humans and cynomolgus monkeys, and identified a number of species-specific features during early embryogenesis. These analyses shed lights on the factors and pathways that are potentially part of the xenogeneic barrier between human and pig, thereby providing critical resources for future studies to develop effective strategies to enhance chimeric contribution of human PSCs in pig embryos.

## Results

### Single-cell dissociation of pig embryos for scRNA-seq analysis

**Fig. 1:**
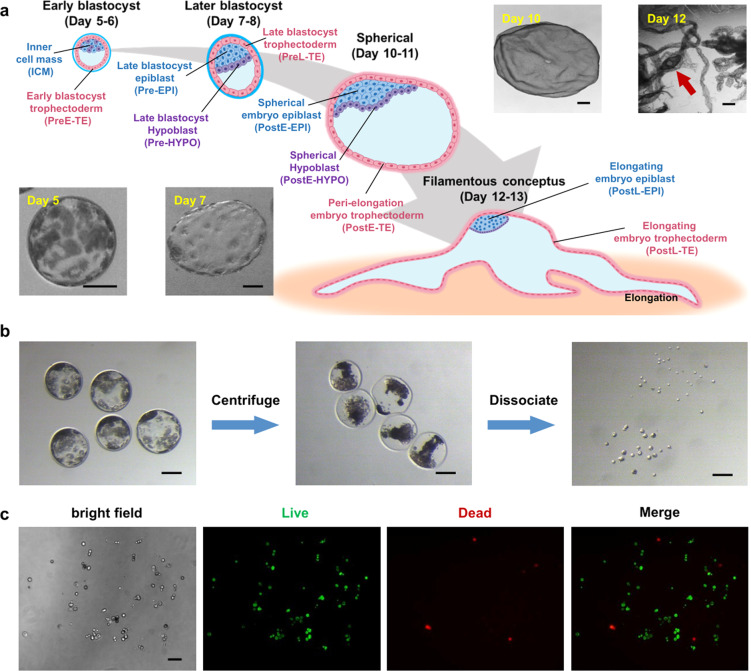
Pig embryo collection and dissociation. **a** Pig embryos collected from four stages for scRNA-seq. **b** Centrifugation and dissociation of pre-implantation pig embryos. **c** Dissociated single cells stained with propidium iodide (PI, red) and 2 μmol/L calcein acetoxymethylester (Calcein-AM, green). Scale bar represents 100 μm.

To study transcriptomic differences between pigs and humans during early embryogenesis, we first sought out to generate a scRNA-seq dataset from pre-gastrulation pig embryos. Efficient single-cell isolation is the first critical step towards successful scRNA-seq experiments. Although a number of studies reported successful dissociation of embryos from non-human primates^18,20^, humans^16,21^ and mice^16,22,23^, difficulty to efficiently dissociate pig blastocysts into single cells impeded scRNA-seq analysis. We first tested several published protocols^16,18,24^ and treated isolated pig inner cell masses (ICMs) with different enzymes, including Tyrpsin, Collagenase IV, Dispase, Pronase, and Hyaluronidase for different durations but found none of them worked efficiently (Supplementary Fig. S1a, b). We next subjected pig blastocysts to a brief centrifugation prior to enzymatic treatment (see materials and methods for details), which led to efficient dissociation of early (E5–6) and late (E7–8) blastocysts into single cells (~22.6 cells/blastocyst) (Fig. 1b, c and Table 1). The method was also efficient in dissociation of embryos at earlier stages (8-cell and morula) (Supplementary Fig. S1e). Besides, we further tried subjecting pig zygotes to a brief centrifugation and treated them with enzymes when they developed to blastocyst, but this method was not efficient and was not applied in the single-cell collection (Supplementary Fig. S1c). With optimized method, we collected single-cell samples from blastocysts and two late pre-implantation stages including spherical and filamentous conceptuses (Supplementary Fig. S1d).

**Table 1:** Statistics of embryos and cells used in this study.

Sample	Description	Number of collected embryos	Number of sequenced cells	Number of selected cells
Day 5	Early blastocyst	8	173	57
Day 7	Late blastocyst	5	121	66
Day 10	Spherical	2	120	96
Day 12	Filamentous conceptus	2	96	82

In total, we isolated 510 single cells from four embryonic stages including early blastocysts (E5–6), later blastocysts (E7–8), spherical (E10–11) and filamentous (E12–13) conceptuses (average 127 cells/each stage) (Fig. 1a and Table 1), and subjected them to library preparation and sequencing. After further quality check that cells with expression of < 3000 genes and outlier cells were excluded (Supplementary Fig. S2a, b), 301 single cells were selected for downstream analysis. Of note is that implantation in pigs start around day 13, so all four stages are technically pre-implantation. To simplify cross-species comparison, herein we designate early blastocysts and later blastocysts as pre-implantation, and spherical and filamentous stages post-implantation.

### Lineage specification in pig embryos

**Fig. 2:**
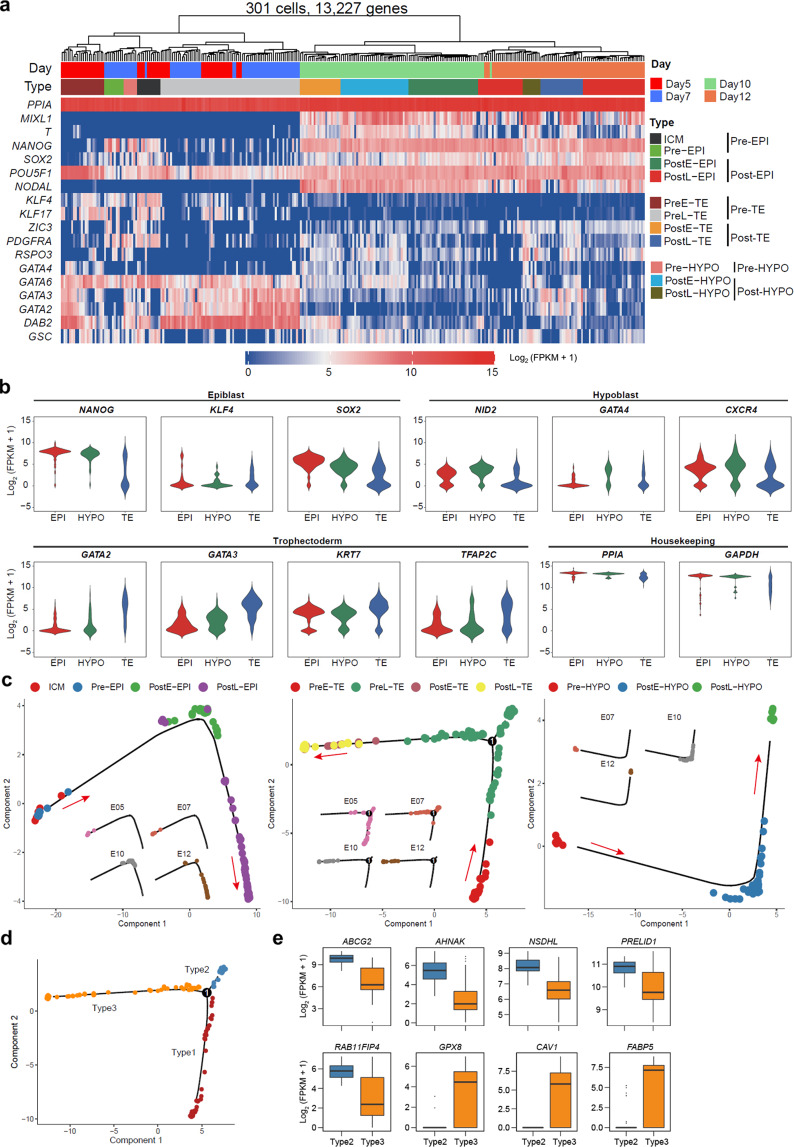
Lineage specification in pig embryos. **a** Unsupervised hierarchical clustering (UHC) with all expressed genes (301 cells, 13,227 genes) and a heatmap of the levels of selected marker genes. Color bars under the dendrogram indicate embryonic days (top) and cell types (bottom), respectively. **b** Violin plots of the expression of selected lineage specifier genes. **c** The development trajectory of each lineage based on psudotime analysis using Monocle2. **d** Cell types defined in the developmental trajectory according to the different branches of TE cells in **c**. **e** Expression patterns of mural TE- and polar TE-related genes in type 2 and 3 TEs. Stage information was indicated as follows: Pre-, Day 5 or/and Day 7; PreE-, Day 5; PreL-, Day 7; Post-, Day 10 and Day 12; PostE-, Day 10; PostL-, Day 12.

Data from all four developmental stages were pooled together and grouped by unsupervised hierarchical clustering (UHC) (13,227 genes) (Fig. 2a). The cells were distinctly classified into two large clusters, which is similar to observation in monkey embryos^18^. According to stage- and lineage-specific markers reported by others^12,17,18,25–28^, cells were further annotated as inner cell mass (ICM), late blastocyst epiblast (Pre-EPI), spherical embryo epiblast (PostE-EPI), elongation embryos epiblast (PostL-EPI), late blastocyst primitive endoderm (Pre-HYPO), spherical embryo hypoblast (PostE-HYPO), elongation embryos hypoblast (PostL-HYPO), early blastocyst trophectoderm (PreE-TE), late blastocyst trophectoderm (PreL-TE), spherical embryo trophectoderm (PostE-TE), and elongation embryos trophectoderm (PostL-TE). Interestingly, the expression level of *POU5F1* was found high in all clusters (Fig. 2a), which is similar to the observations in cynomolgus monkey^18^ and human embryos^29^. Our results confirmed that *KLF4* was highly expressed in ICM cells (Fig. 2a), while significantly downregulated in EPI as embryos further develop^12,18^. It was previously reported that *KLF4* was also expressed in mural TE but not polar TE in mouse embryos^30^. Similarly, in addition to ICM, we found some PreE-TEs expressed *KLF4* (Fig. 2a, b), suggesting their mural TE identity. We found *NODAL* was expressed in cells from spherical and filamentous conceptuses, but not blastocysts (Fig. 2a), which is consistent with a previous report^12^. *GATA6*, a hypoblast marker gene, was found highly expressed in blastocysts, and PostE- and PostL-HYPO of spherical and filamentous conceptuses, respectively (Fig. 2a).

To independently confirm proper lineage annotation, we pooled the same cell types together (ICM/EPI, HYPO or TE) and examined the expression of known lineage markers. As a control, we found the housekeeping gene *PPIA* was expressed at comparable levels in cells from all three lineages (Fig. 2a, b). We found cells annotated as EPI, TE and HYPO correctly expressed their respective lineage markers (Fig. 2b). Pseudotime analysis revealed that temporal progression of each annotated lineage was in accordance with the development time of embryos we sampled (Fig. 2c). The bifurcation in PreL-TEs suggests two sub-types of cells are formed. The TE of mouse late blastocyst is subdivided into polar TE, which covers the epiblast at the embryonic pole, and mural TE, which overlays the blastocyst cavity at the abembryonic pole^31,32^. We thus analyzed the expression levels of polar and mural TE-associated genes as previously reported^19,30,33^. We found TE genes such as *ABCG2*, *AHANK*, *NSDHL*, *PRELID1*, *RAP11FIP4* were highly expressed in type 2 TEs, and polar TE-related genes such as *FABP5*, *CAV1* and *GPX8* were highly expressed in type 3 TEs (Fig. 2d, e), suggesting type 2 and 3 TEs likely correspond to mural and polar TEs, respectively. In addition, three-dimensional (3D) principal component analysis (PCA) plots showed that ICM/EPI, HYPO, and TE cells were roughly divided into two main clusters, day 5/day 7 and day 10/day 12, indicating a more dramatic change occurred between day 7 and day 10 of pig pre-gastrulation development (Supplementary Fig. S2c).

Taken together, our scRNA-seq dataset identified cells from all three founder tissues spanning four pre-gastrulation developmental stages, constituting the most complete single-cell landscape of pig early development to date.

### Cross-species comparison of EPI development and pluripotency progression

**Fig. 3:**
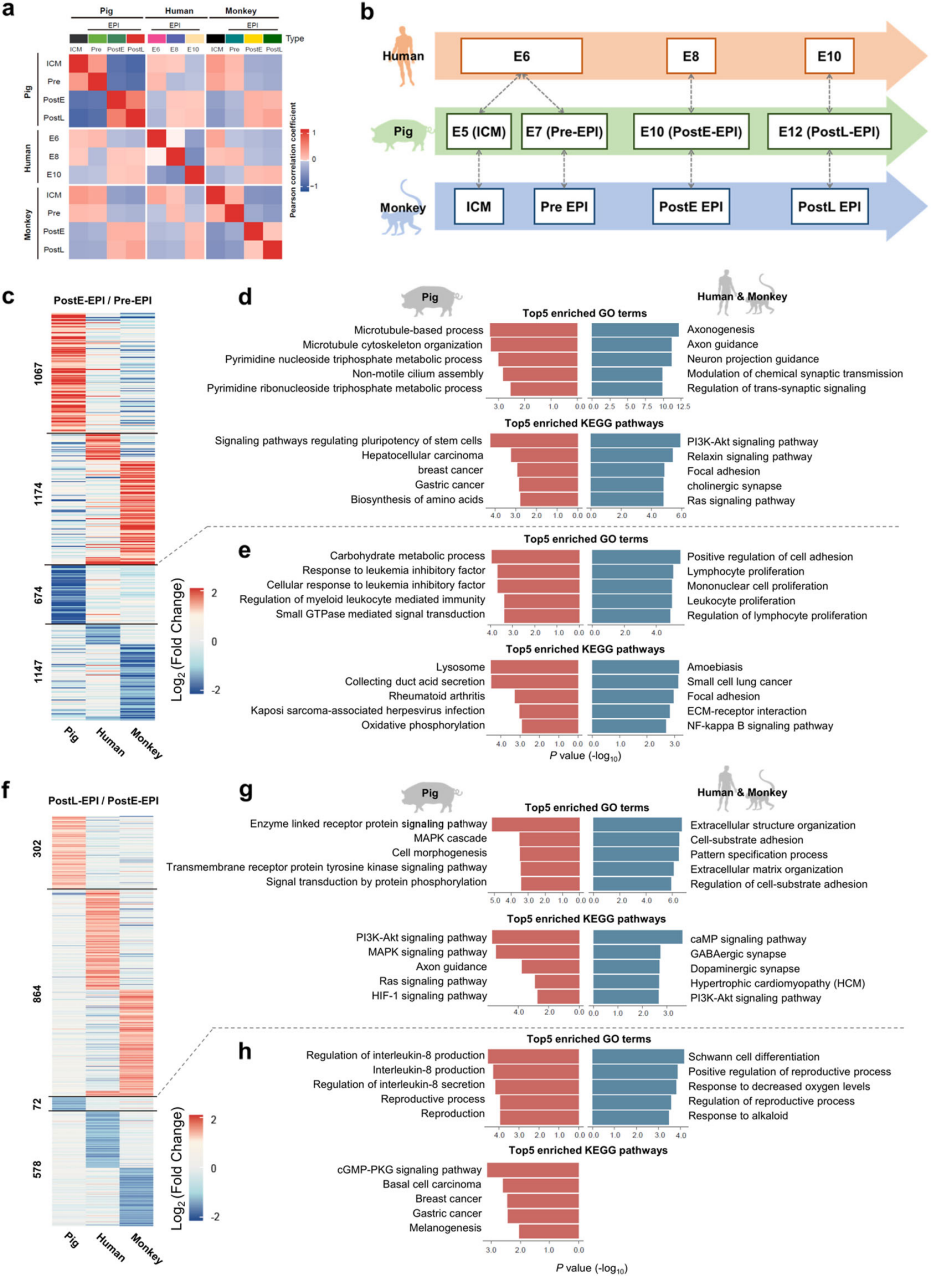
Cross-species comparison of EPI development and pluripotency progression. **a** Heatmap of the correlation coefficients among EPIs. **b** Schematic diagram showing developmental coordinate of the spectrum of human, monkey and pig pluripotency. **c** Heatmap of DEGs of Pre- and PostE-EPIs in pigs, humans, and monkeys. **d** Top 5 enriched GO terms and KEGG pathways of upregulated DEGs of Pre- and PostE-EPIs in each species. **e** Top 5 enriched GO terms and KEGG pathways of downregulated DEGs of Pre- and PostE-EPIs in each species. **f** Heatmap of DEGs of PostE- and PostL-EPIs in pigs, humans, and monkeys. **g** Top 5 enriched GO terms and KEGG pathways in upregulated DEGs of PostE- and PostL-EPIs in each species. **h** Top 5 enriched GO terms and KEGG pathways in downregulated DEGs of PostE- and PostL-EPIs in each species. Stage information was indicated as follows: Pre-, Day 5 or/and Day 7; PreE-, Day 5; PreL-, Day 7; Post-, Day 10 and Day 12; PostE-, Day 10; PostL-, Day 12.

EPI is a single-cell-layered epithelium that gives rise to all tissues in an adult body. Successful human–pig chimera formation will depend on the survival, proliferation, and proper differentiation of human PSCs within the EPI layer of the pig embryos. Therefore, cross-species comparisons of EPI transcriptomes at different developmental stages may help identify species-specific features underlying the xenogeneic barrier. Here, we performed comparative transcriptome analysis using two published scRNA-seq datasets from human and cynomolgus monkey (herein referred to as monkey) embryos, together with our pig dataset^18,19^. To minimize the bias of stage mismatching across species, we first performed Pearson correlation analysis comparing EPIs from different developmental stages and species (Fig. 3a). We found human E10 EPIs showed higher correlation coefficients with both pig and monkey PostL-EPIs than other stages; human E8 EPIs was more similar to pig and monkey PostE-EPIs; and human E6 EPIs correlated better with pig and monkey ICMs and Pre-EPIs (Fig. 3b). Furthermore, from 3D PCA plots both monkey and pig EPIs were found divided into two main clusters: ICM/Pre-EPIs and PostE-EPIs/PostL-EPIs, but this is not the case with human EPIs (Supplementary Fig. S3a). This is likely due to the source of embryos used for scRNA-seq analysis: monkey and pig embryos were obtained in vivo while human embryos were cultured in vitro.

We first compared early EPI development among pig (ICM/Pre-EPI to PostE-EPI), monkey (Pre-EPI to PostE-EPI) and human (E6 to E8). Heatmap of differentially expressed genes (DEGs) revealed that 1067 and 1174 genes were upregulated while 674 and 1147 genes were downregulated in pig and human/monkey, respectively (Fig. 3c; Supplementary Table S1). Gene ontology (GO) term and Kyoto Encyclopedia of Genes and Genomes (KEGG) pathway enrichment analyses further showed that genes upregulated in pig were enriched in terms such as “signaling pathways regulating pluripotency of stem cells”, “pyrimidine metabolic process” and “microtubule-based process” (Fig. 3d; Supplementary Table S1). In contrast, genes upregulated in human/monkey were enriched in “PI3K-Akt signaling pathway”, and neuron development, including “axonogenesis” and “neuron projection guidance” (Fig. 3d; Supplementary Table S1), which agrees with a recent report^18^. Genes downregulated in pig were mainly involved in carbohydrate metabolic process and leukemia inhibitory factor (LIF)-mediated signal transduction, while those downregulated in human/monkey were enriched in several terms related to the proliferation of immune cells (Fig. 3e).

Next, we compared late EPI development among pig (PostE-EPI to PostL-EPI), monkey (PostE-EPI to PostL-EPI) and human (E8 to E10). Heatmap of DEGs showed that 302 and 864 genes were upregulated, and 72 and 578 genes were downregulated in pig and human/monkey, respectively (Fig. 3f; Supplementary Table S1). GO term and KEGG pathway enrichment analyses further revealed that genes specifically upregulated in pig were involved in enzyme-linked receptor protein signaling pathway and cell morphogenesis, while those in human/monkey were related to extracellular structure organization and cell junction assembly (Fig. 3g). Genes downregulated in pig were mainly involved in IL8 production and secretion, and reproductive process, while those in human/monkey were enriched in response to decreased oxygen levels (Fig. 3h). Interestingly, while the PI3K-Akt pathway was enriched with upregulated genes in human/monkey early EPI development, it was overrepresented in genes upregulated during pig late EPI development (Fig. 3d, g). However, specific targets in the PI3K-Akt pathway exhibited different expression patterns between pig and human/monkey (Supplementary Fig. S3b). For example, *ITGA5* was significantly upregulated in human/monkey, but downregulated in pig.

**Fig. 4:**
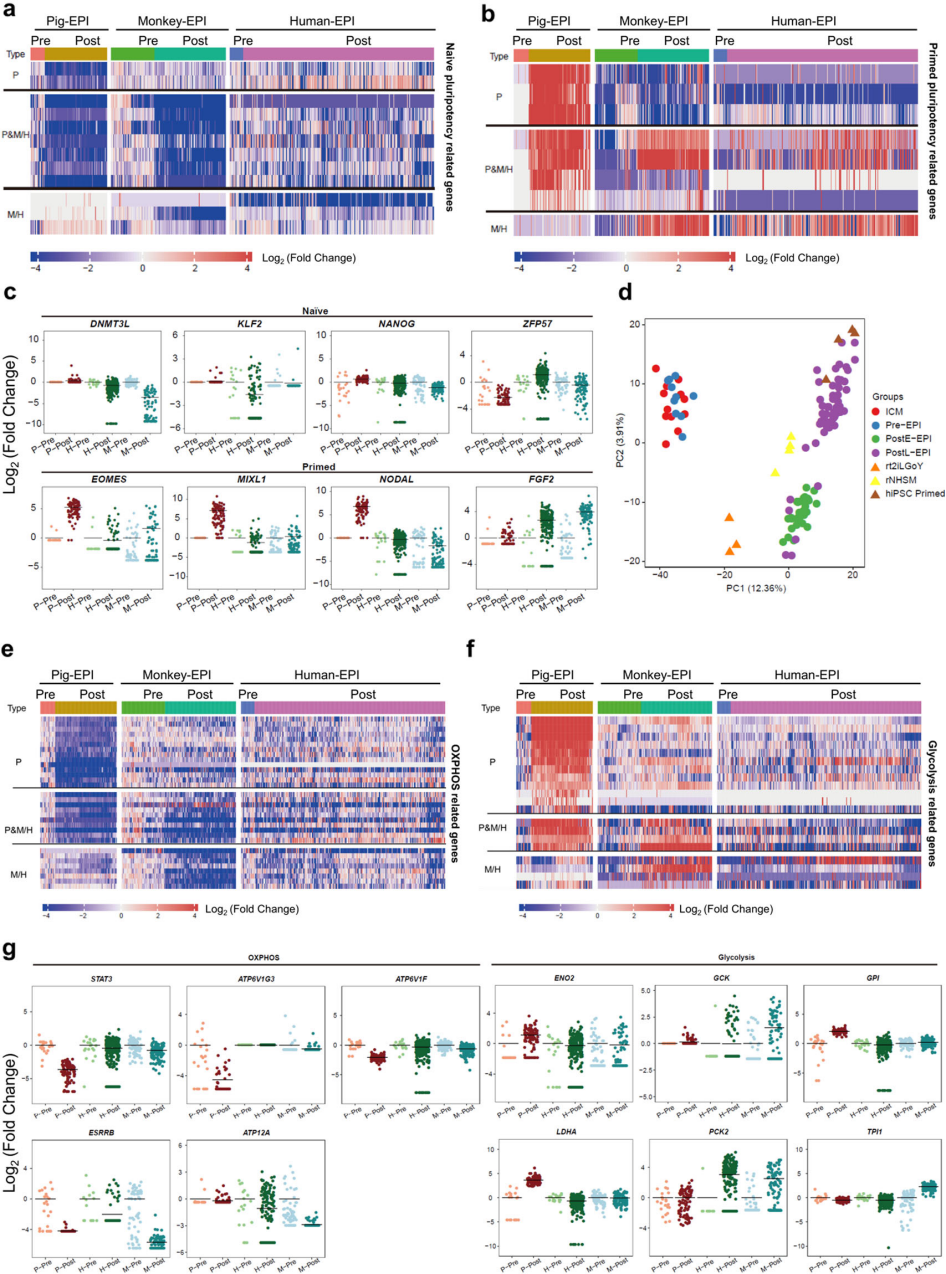
Cross-species comparison of metabolic, epigenetic, and transcriptional regulations of pluripotency among pig, monkey, and human EPIs. **a** Heatmap of naïve-related genes in Pre- and Post-EPIs in pigs, humans, and monkeys. **b** Heatmap of primed pluripotency-related genes in Pre- and Post-EPIs in pigs, humans, and monkeys. **c** Scatter-plot of species-specific naïve and primed pluripotency-related genes. P-Pre, pig ICM and Pre-EPIs; P-Post, pig PostE- and PostL-EPIs; H-Pre, human D6-EPIs; H-Post, human D8- and D10-EPIs; M-Pre, monkey ICM and Pre-EPIs; M-Post, monkey PostE- and PostL-EPIs. Using Pre-EPIs as a control, fold change of gene expression in each cell relative to the average expression level in Pre-EPIs was calculated. **d** PCA of pig EPIs and human iPSCs. **e** Heatmap of OXPHOS-related genes in Pre- and Post-EPIs in pigs, humans, and monkeys. **f** Heatmap of glycolysis-related genes in Pre- and Post-EPIs in pigs, humans, and monkeys. **g** Scatter-plot of species-specific OXPHOS- and glycolysis-related genes. Stage information was indicated as follows: Pre-, Day 5 or/and Day 7; PreE-, Day 5; PreL-, Day 7; Post-, Day 10 and Day 12; PostE-, Day 10; PostL-, Day 12. The horizontal lines in **c** and **g** indicate the mean values. To avoid the influence caused by the “0” value, a new matrix was generated by using FPKM + 1 and the log_2_-transformed fold change was shown.

EPI development is accompanied by naïve-to-primed pluripotency transition, which may show different dynamics between species. Next, we performed cross-species comparison of transcriptome changes during naïve-to-primed pluripotency transition. We pooled data from human, monkey and pig early and late blastocysts together (Pig ICM and Pre-EPI; monkey ICM and Pre-EPI;^18^ human E6 EPI^19^), which are collectively labeled as Pre-EPIs for simplicity. We also pooled data from human and monkey post-implantation stages, and pig spherical and filamentous conceptuses together (Pig PostE-EPI and PostL-EPI; monkey PostE-EPI and PostL-EPI;^18^ human E10 EPI^19^), which are referred to as Post-EPIs. We found that, in general, naïve pluripotency-related genes were downregulated (Fig. 4a; Supplementary Fig. S4a, Tables S2 and S3) while primed pluripotency-related genes were upregulated in Post-EPIs versus Pre-EPIs from all three species (Fig. 4b; Supplementary Fig. S4b, Tables S2 and S3). However, expression patterns of some pluripotency marker genes differed among species. For example, naïve genes such as *STAT3* and *ZFP57* were significantly downregulated in pig but not in monkey and human Post-EPIs. In contrast, *KLF2*, *DNMT3L* and *NANOG* were significantly downregulated in monkey and human but not in pig Post-EPIs. Primed genes such as *EOMES*, *MIXL1* and *NODAL* were only upregulated in pig while *FGF2* was only upregulated in monkey and human (Fig. 4c). Next, we included several naïve and primed human iPSCs^34^ (rt2iLGoY-hiPSCs, rNHSM-hiPSCs and primed hiPSCs) for comparison. Pearson correlation analysis revealed that rt2iLGoY-hiPSCs resembled pre-implantation human E6 EPIs and primed hiPSCs resembled post-implantation human E10 EPIs. Interestingly, we observed NHSM-hiPSCs were closer to E8 rather than E6 and E10 human EPIs, which is intermediate between rt2iLGoY-hiPSCs and primed hiPSCs (Supplementary Fig. S4c). Similarly, PCA analysis placed rt2iLGoY-hiPSCs between pig ICM/Pre-EPIs and PostE-EPIs, NHSM-hiPSCs between PostE-EPIs and PostL-EPIs, and primed hiPSCs closer to pig PostL-EPIs (Fig. 4d).

In summary, we have systematically compared EPI development among pig, human and monkey. Our results reveal the divergence in a number of pathways involved in early and late EPI transitions and identify several pig and primate (human and monkey)-specific pluripotency features. These findings also identify a developmental coordinate of pluripotency spectrum among pig, human and monkey.

### Cross-species comparison of metabolic, epigenetic, and transcriptional regulations of pluripotency

Extensive metabolic changes were observed during early embryo development from different species^16,35–37^. A developing embryo transits through stages with rapidly changing anabolic and catabolic demands, which necessitates evolving of the metabolic infrastructure and associated pathways to match these demands. To identify species-specific metabolic regulations of pluripotency, we mined the scRNA-seq datasets from pig, human and monkey, and compared the expression levels of genes involved in oxidative phosphorylation (OXPHOS) and glycolysis. In agreement with a previous report^12^, we observed that genes involved in OXPHOS were downregulated (Fig. 4e; Supplementary Fig. S4d and Table S3), while genes related to glycolysis were upregulated in Post-EPIs versus Pre-EPIs in both pig and monkey (Fig. 4f; Supplementary Fig. S4e and Table S3). Of note is that similar changes in human EPIs were less obvious, which is likely due to the use of embryos cultured in vitro. Although the overall trends in metabolic changes were similar, the expression patterns of certain genes differed among species. For example, OXPHO-related genes such as *STAT3*, *ATP6V1F*, and *ATP6V1G3* were only found downregulated in pig Post-EPIs, and *ATP12A* was specifically downregulated in human/monkey Post-EPIs (Fig. 4g). We also found *ESRRB* expression level decreased to a greater extent in monkey than in pig and human Post-EPIs (Fig. 4g). Species-specific expression patterns of genes involved in glycolysis were also observed, e.g., *ENO2*, *GPI*, and *LDHA* expression levels increased only in pig Post-EPIs, while *GCK*, *PCK2*, and *TPI1* were specifically upregulated in monkey and human Post-EPIs (Fig. 4g).

Metabolism is intrinsically linked with epigenetics, as many chromatin modifications are generated with substrates derived from different metabolic pathways^38^. Recent studies also point to a link between metabolism and the epigenetic control of pluripotent states^39^. We compared the expression patterns of genes involved in epigenetic regulations during EPI development among pig, human and monkey. We observed that de novo DNA methylase DNMT3A was significantly upregulated in human and monkey but not pig Post-EPIs. In contrast, DNA demethylase TET2^40^ was downregulated to a greater degree in monkey and human than in pig Post-EPIs (Supplementary Fig. S4f). Previous reports have indicated that different components of PRC2, a multiprotein enzyme complex that catalyzes histone H3 tri-methylation (H3K27me3), show distinct functions. Knockdown of *Ezh2* but not *Ezh21* affects global H3K27me2/3 levels in mouse embryonic fibroblasts^41^. A recent study also reported that hESCs showed distinct morphology upon deletion of different PRC2 components^42^. Interestingly, our analysis revealed distinct expression patterns of PRC2 complex components between species: (1) we found *EZH2* was downregulated in pig but upregulated in monkey Post-EPIs; (2) more pronounced upregulation of *EZH1* was found in human and monkey Post-EPIs than pig; (3) *SUZ12* and *EED*, on the other hand, were found more dramatically downregulated in monkey and pig Post-EPIs, respectively (Supplementary Fig. S4f). *SUZ12*, *EED*, and *EZH2* did not exhibit any significant change between Post-EPIs and Pre-EPIs in cultured human embryos (Supplementary Fig. S4f).

We also investigated species differences in the expression patterns of transcription factors (TFs) and cofactors during EPI development. By filtering DEGs (Pre-EPIs versus Post-EPIs) through the TF and cofactor data in Animal TFDB (http://bioinfo.life.hust.edu.cn/AnimalTFDB/), we found that *MBD*, *NF-YA* and *STAT* TF families were enriched in pig Pre-EPIs, while homeobox TF family was specifically and highly expressed in monkey and human Pre-EPIs (Supplementary Fig. S4h). In addition, we also observed that *GCM*, *COE*, *CUT* and *THAP* TF families were overrepresented in monkey and human Post-EPIs (Supplementary Fig. S4h). Genes of cofactor families including nucleoplasmin, *PCGF*, *PHF*, and *SET* were enriched in pig Post-EPIs, while “Other CRF Vestigial like” cofactor families were highly expressed in human and monkey Post-EPIs (Supplementary Fig. S4g).

Taken together, these results demonstrate overall similar expression patterns of OXPHOS- and glycolysis-related genes among pig, human and monkey EPIs, and identified a number of species-specific epigenetic regulators and TFs/cofactors during EPI development.

### Cross-species comparison of membrane-related genes among pig, monkey, and human EPIs

**Fig. 5:**
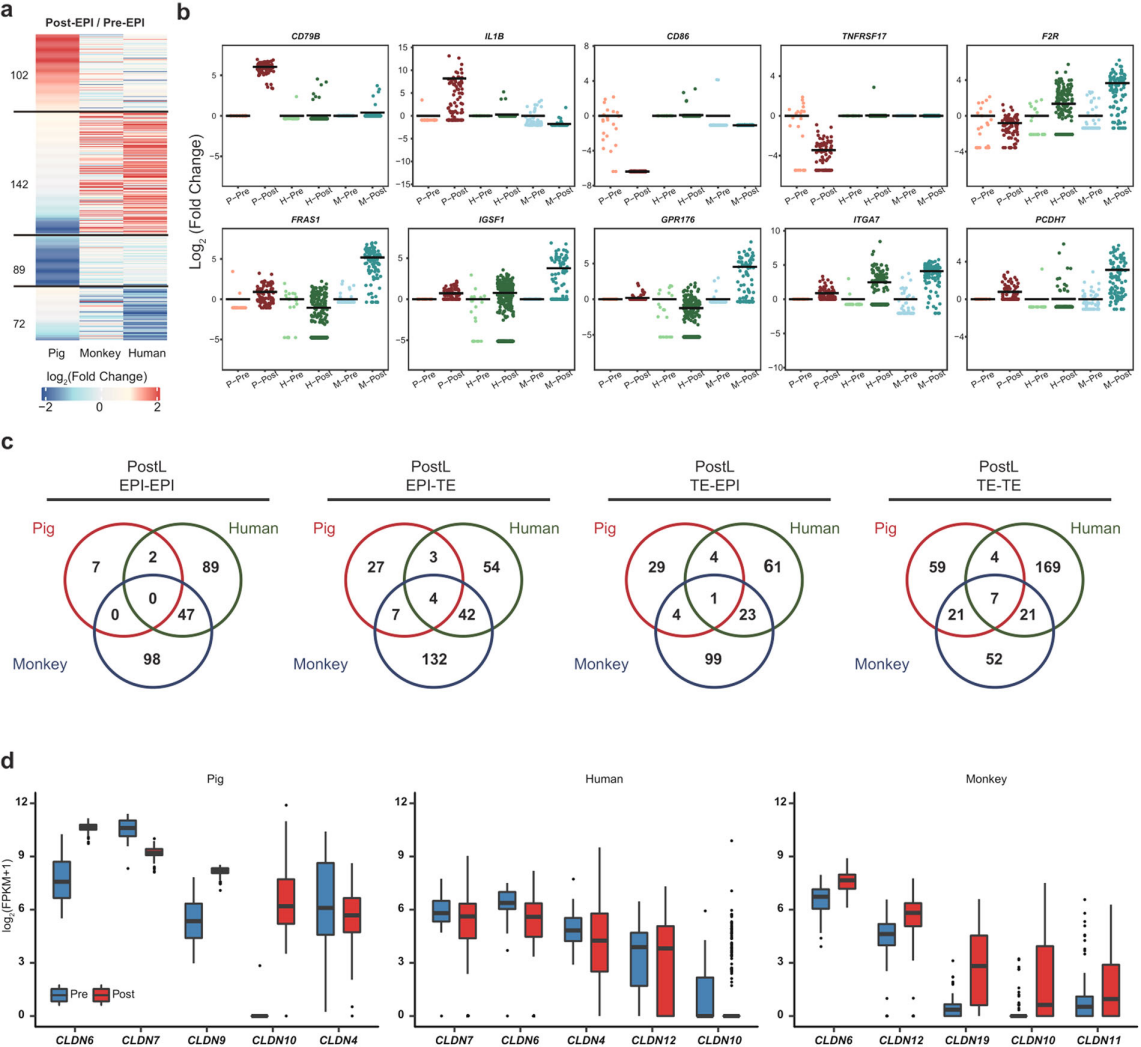
Cross-species comparison of membrane-related genes among pig, monkey and human EPIs. **a** Heatmap of differential expression of cell surface proteins in Pre- and Post-EPIs in pig, human, and monkey. **b** Scatter-plot of genes encoding species-specific surface proteins. P-Pre, pig ICM and Pre- EPIs; P-Post, pig PostE- and PostL- EPIs; H-Pre, human D6-EPIs; H-Post, human D8- and D10-EPIs; M-Pre, monkey ICM and Pre-EPIs; M-Post, monkey PostE- and PostL-EPIs. Using Pre-EPIs as a control, fold change of gene expression in each cell relative to the average expression level in Pre-EPIs was calculated. **c** Overlaps of the interaction relationship between PostL-EPIs and PostL-TEs in each species. **d** Box plot showing the expression of top 5 claudin genes in Pre- and Post- EPIs in each species. Stage information was indicated as follows: Pre-, Day 5 or/and Day 7, PreE-, Day 5; PreL-, Day 7; Post-, Day 10 and Day 12; PostE-, Day 10; PostL-, Day 12. The horizontal lines in **b** indicate the mean values. To avoid the influence caused by the “0” value, a new matrix was generated by using FPKM + 1 and the log_2_-transformed fold change was calculated.

Interspecies cell adhesion and/or ligand–receptor incompatibilities could be among the causes of low interspecies chimera formation efficiency^4^. Thus, identification of putative incompatible receptors and cell adhesion molecules may provide useful information to help overcome the xenogeneic barrier. Here, we compared the expression patterns of membrane-related genes among pig, monkey and human EPIs. Heatmap based on the expression levels of 405 membrane-related genes showed a similar pattern between human and monkey, which is different from that of pig (Fig. 5a). Among these genes, we identified 102 were specifically upregulated during pig ICM/Pre-EPI to Post-EPI transition. Similarly, 142 genes were upregulated during corresponding stage transitions in monkey and human (Fig. 5a; Supplementary Table S4). Interestingly, surface receptors such as immune- and inflammatory-related genes *CD86* and *TNFRSF17* were downregulated in pig Post-EPIs when compared to ICM/Pre-EPIs (Fig. 5b). In contrast, no change in these genes was observed in human and monkey (Fig. 5b). Previous studies demonstrated that the implantation process is associated with maternal immune response to the allogeneic fetus and inflammation^14,43,44^. Our results suggest different immune- and inflammatory-related genes may facilitate the implantation process in pig than human/monkey embryos. G-protein-coupled receptor activity-related genes including *F2R* and *GPR176* were upregulated in monkey but not pig Post-EPIs (Fig. 5b). In addition, pig primed pluripotency cell surface marker *CD79B*^12^ was found specifically expressed in pig Post-EPIs (Fig. 5b), which is consistent with a recent report^12^.

**Fig. 6:**
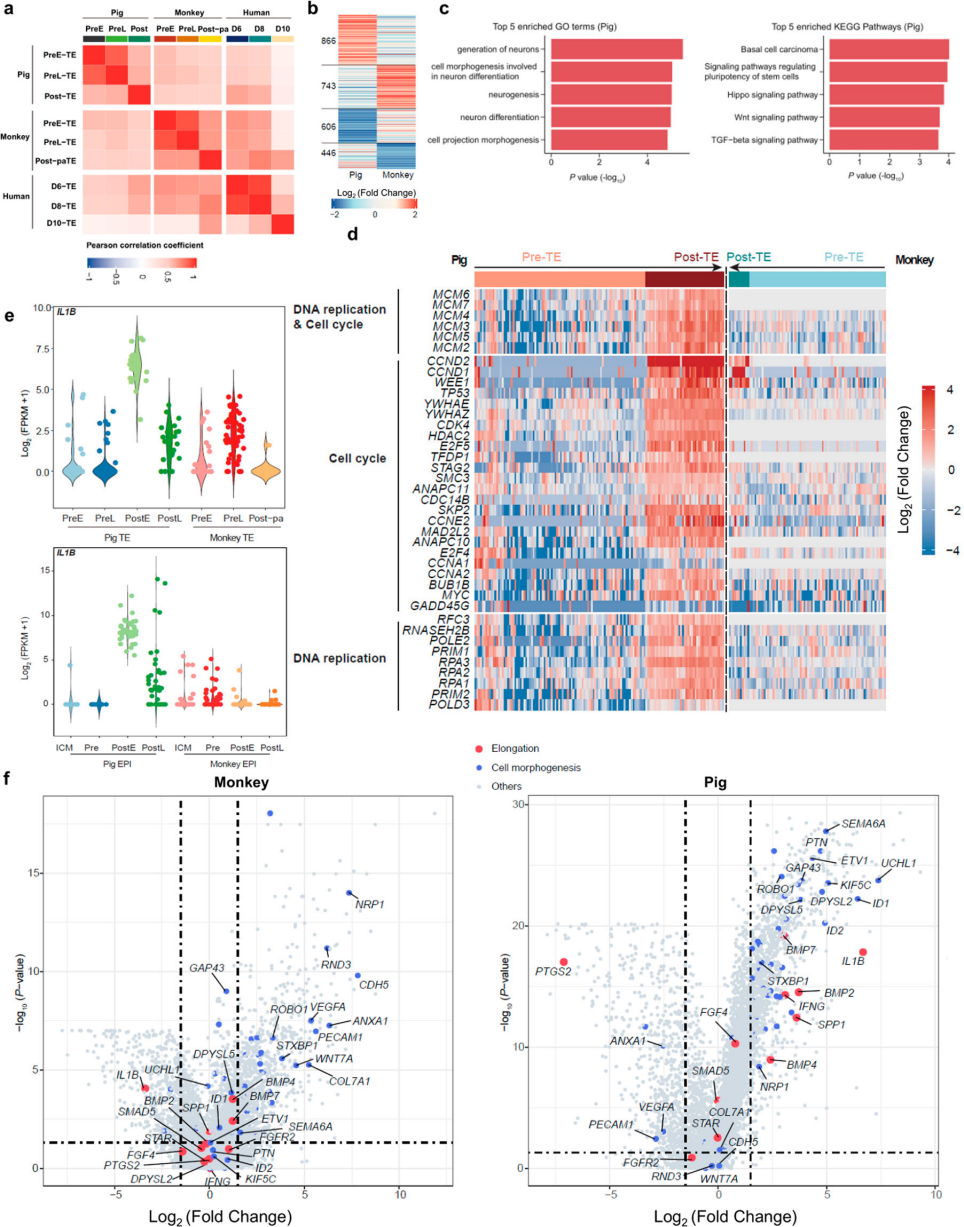
Cross-species comparison of TE development. **a** Heatmap of the correlation coefficients among TEs in pig, human, and monkey. **b** Heatmap of DEGs of Pre- and PostE-TEs in pigs and monkeys. **c** Top 5 enriched GO terms and KEGG pathways of upregulated DEGs in pig Pre- and PostE-TEs. **d** Heatmap of fold change of cell cycle- and DNA replication-related genes in Pre- and Post-TEs of pigs and monkeys. **e** Violin plots of the expression of *IL1B* in pig and monkey EPIs and TEs from different stages. **f** Volcano plot showing DEGs between Pre- and Post-TEs of monkey and pig, respectively. Elongation- and cell morphogenesis-related genes are shown in red and blue, respectively; others are shown in gray. Stage information was indicated as follows: Pre-, Day 5 or/and Day 7; PreE-, Day 5; PreL-, Day 7; Post-, Day 10 and Day 12; PostE-, Day 10; PostL-, Day 12.

Among cell adhesion-related proteins, IL1B was upregulated in pig Post-EPIs, while *FRAS1*, *ITGA7* and *PCDH7* were upregulated in monkey Post-EPIs (Fig. 5b; Supplementary Table S4). As *IL1B* has been previously suggested to play an important role in pig early embryogenesis, we further checked its expression in all four stages and found that *IL1B* expression levels peaked at day 10 (PostE-EPIs) (Fig. 6e). We also performed cell communication analysis and identified two interactions uniquely related to *IL1B* in pig Post-EPIs including IL1 receptor_IL1B and IL1B_ADRB2 when compared with monkey and human Post-EPIs (Supplementary Fig. S5a and Table S5). In addition, interactions including CCL3_IDE and BDNF_SORT1 were uniquely found in pig Post-EPIs (Supplementary Fig. S5a and Table S5). We next performed the analysis of cell–cell communication between the human PSCs (naïve state: N-PSC; intermediated state: I-PSC; primed state: P-PSC) and pig EPIs at different stages (E5, E7, E10 and E12). We found that EPI-PSCs/PSCs-EPI shared 59%–82% of the same cell–cell interactions as EPI–EPI at different stages (Supplementary Fig. S5e and Table S6). The overall rate of overlapped cell–cell interactions did not show significant difference among 3 types of PSCs with pig EPIs, but each types of PSCs showed their specific cell–cell interaction with different stages of pig EPIs (Supplementary Fig. S5e and Table S6).

Lack of intercellular communication between species may represent one of the barriers to interspecies chimerism. To identify potential interspecies incompatibility in cell–cell communication, we used CellPhoneDB^45^ to predict the crosstalk between PostL-EPI and PostL-TE in pig, human, and monkey. We compared ligand–receptor interactions between different cell types among different species. Our results revealed that: (1) 7, 89, and 98 pairs were predicted uniquely between Post-EPIs (EPI–EPI) in pig, human, and monkey, respectively; (2) 59, 52, and 169 pairs were predicted uniquely between Post-TEs (TE–TE) in pig, human, and monkey, respectively; (3) 27, 54, and 132 pairs were predicted uniquely between Post-EPIs and Post-TEs (EPI–TE) in pig, human, and monkey, respectively; and (4) 29, 61, and 99 pairs were predicted uniquely between Post-TEs and Post-EPIs (TE–EPI) in pig, human, and monkey, respectively (Fig. 5c). In general, we found there were more shared interaction pairs between human and monkey than those between human and pig, and monkey and pig (Fig. 5c). Next, we performed GO enrichment and KEGG pathway analyses to determine putative functions of pig-specific ligand–receptor interactions (Supplementary Fig. S5c, d and Table S5). These analyses revealed that unique interactions in pig were more enriched in TE–TE, to a lesser extent in EPI–TE and TE–EPI, and not much in EPI–EPI (Supplementary Fig. S5c, d and Table S5). GO analysis showed that putative TE–TE unique interactions were enriched in terms related to cell adhesion, proliferation and tube morphology function, indicating trophoblasts undergo significant cellular changes during spherical and filamentous stages (Supplementary Fig. S5c and Table S5). KEGG pathway analysis showed that the PI3K-Akt signaling pathway was overrepresented in TE–EPI and TE–TE interactions, while the TGF-beta signaling pathway and signaling pathways regulating pluripotency of stem cells were more enriched in EPI–TE and TE–TE interactions (Supplementary Fig. S5d and Table S5).

Mammalian early embryogenesis is accompanied by epiblast epithelialization and the formation of tight junctions between neighboring cells. Next, we performed comparative analysis of cell junction-related genes across species, with a specific focus on the claudin family that are the main constituents of the tight junction complexes^46^. We found both *CLDN4* and *CLDN6* were expressed at higher levels than other claudin family members in all three species (Fig. 5d), suggesting conserved roles of these two claudin genes during early mammalian development. In contrast, *CLDN9* and *CLDN10* were exclusively upregulated in pig Post-EPIs versus Pre-EPIs. These differentially expressed claudins may prevent tight junction formation between donor human PSCs with pig EPI, and thereby constituting a potential barrier for efficient chimera formation.

Taken together, these analyses uncover differences in expression patterns of genes related to surface receptors, cell adhesion and tight junctions between pig and human/monkey EPIs. These differences may prevent proper recognition and communication between donor human/monkey cells and cells from host pig embryos, resulting in low integration and survival of donor cells in cross-species chimera.

### Cross-species comparison of TE development

Although several key signaling pathways to initiate uterine receptivity are conserved, embryo implantation differs across species^47^. Different from primates with hemochorial placentas that have an “invasive” implantation process, pig has epitheliochorial placentas, which exhibit a “non-invasive” implantation process^43,47^. During implantation, through trophoblast proliferation and organization, pig conceptuses elongate to establish pregnancy^14^. This elongation process is absent in primates, which may negatively affect human PSC viability, proliferation, and differentiation within pig embryos. It is well-established that extraembryonic tissues not only are necessary for nutrition and regulating implantation during development, but also play crucial roles in patterning the embryo before and during gastrulation^48^. For example, the extraembryonic ectoderm signals to the proximal epiblast, inducing expression of several genes important for mouse embryo posterior proximal identity, via BMP4 and BMP8b signaling^49,50^. In addition, Nodal (Lefty1 and Cer1) and the Wnt (Dkk1) pathway antagonists expressed from extraembryonic tissues play important roles in mouse embryonic patterning^51,52^. Thus, we hypothesize that miscommunication between donor human cells and host pig extraembryonic tissues may also impede the chimera formation. To gain insights into species-specific trophoblast development, we performed cross-species transcriptome comparison of TE lineages at different developmental stages (Pre-TE and Post-TE) among pig, human, and monkey. Pearson correlation analysis showed lower correlation coefficients between pig Post-TEs and human/monkey Post-TEs than between human and monkey Post-TEs (Fig. 6a), suggesting that monkey Post-TEs are more similar to human Post-TEs. Similar to EPIs, 3D PCA analysis showed that both monkey and pig TEs, but not human TEs, were divided into two main clusters: PreE-TEs/PreL-TEs and PostE-TEs/PostL-TEs (Supplementary Fig. S6a, b), which is likely reflective of different embryo sources: in vivo (pig and monkey) versus in vitro (human). To avoid the influences caused by in vitro culture, here we only performed cross-species comparison of Pre-TE to Post-TE transition between monkey and pig. Our analysis identified a large number of DEGs, including 866 and 743 specifically upregulated in pig and monkey, respectively (Fig. 6b; Supplementary Table S7). GO term enrichment and KEGG pathway analyses revealed that genes upregulated in pig Post-TEs were enriched in terms related to neurogenesis, several developmental pathways, including the Hippo, WNT, and TGF-beta signaling pathways, and microtubule-related processes (Fig. 6c). In contrast, genes specifically upregulated in monkey Post-TEs were enriched in terms related to migration and angiogenesis (Supplementary Table S7). GO term enrichment analysis also revealed that cell cycle and DNA replication pathway-related genes were uniquely upregulated in pig Post-TEs versus Pre-TEs (Fig. 6d; Supplementary Table S7), suggesting rapid cell proliferation may contribute to pig TE development. In contrast, there was no obvious change in the expression levels of these genes during monkey TE development (Fig. 6d). To confirm these changes are specific to pig TE development, we further examined the expression patterns of these cell cycle- and DNA replication-related genes in pig and monkey EPIs. Results revealed that these genes also exhibited species-specific upregulation in pig Post-EPIs (Supplementary Fig. S6c).

Rapid embryo elongation during the process of pregnancy establishment is unique to pig when compared to human/monkey, however, the underlying mechanisms remain unclear. To gain molecular insights into the pig elongation process, we examined DEGs between Pre-TEs and Post-TEs in both monkey and pig. Our results showed that genes such as *IL1B*, *BMP2/4/7*, *SPP1*, *SMAD5*, *FGF4*, and *FGFR2*^14,15,53,54^, which have been implicated in regulating embryo elongation, were upregulated in pig Post-TEs, but not in monkey Post-TEs (Fig. 6f). Interestingly, we found *IL1B*, a gene reported to be essential for the elongation of pig embryo^54^ was markedly upregulated in pig Post-TEs but downregulated in monkey Post-TEs when compared to Pre-TEs (Fig. 6e, f). The expression of *IL1B* peaked in TEs of spherical conceptus and then gradually decreased, which is consistent with previous reports^15,55^. Interestingly, a similar expression pattern of *IL1B* could be found in pig EPIs (Fig. 6e). Next, we confirmed IL1B target genes, such as *IFNG*, *IL8*, *Spi1*, *MIB1*, were also significantly upregulated during Pre-TE to Post-TE transition (Supplementary Fig. S6d and Table S8). Among these IL1B target genes, *IFNG* (Type II *IFN*) and *IL8-*related NFKB signaling were suggested to be required for successful implantation and endometrial functions in pigs^14,44^. In addition, we also analyzed species-specific interactions between PostL-TEs and PostL-TEs. The ligand–receptor pairs such as MDK_SORL1, MDK_LRP1, MDK_PTPRZ1, EFNA1_EPHA4, EFNA1_EPHA7, CXADR_FAM3C, and CER1_MRC2 were uniquely presented in pig PostL-TEs and PostL-TEs interactions (Supplementary Fig. S5b), which were enriched in GO terms including cell adhesion, biological adhesion, cell–cell adhesion, tube morphogenesis (Supplementary Fig. S5c and Table S5). These biological processes and pathways have been previously suggested to play important roles in pig embryo elongation and implantation^14^.

It has been reported that cadherins could affect axon outgrowth and elongation^56–58^. We then investigated cadherin-related genes in both pig and monkey TEs. Our results showed that *CDH2*, *CDH7*, and *CDH11* were uniquely upregulated in pig Post-TEs, while *CDH1* and *CDH5* were specifically downregulated and upregulated in monkey Post-TEs, respectively (Supplementary Fig. S6e). Interestingly, both *CDH2* and *CDH11* have been previously reported to be involved in axon elongation^56,57^. These results suggest that pig embryo elongation during pre-implantation development shares some molecular characteristics of axon elongation.

To investigate potential roles of the endometrial epithelium may play in pig embryo elongation, we used CellPhoneDB^45^ to predict the ligand–receptor interactions between pregnant womb epithelium (luminal and glandular, P_LE and P_GE) and PostL-EPIs or PostL-TEs during the elongation stages. We identified 420 pairs of potential ligand–receptor interaction pairs in pregnant womb (Supplementary Table S9). Of which, 18 ligand–receptor interaction pairs were specifically identified in the pregnant womb when compared with the none-pregnant control (Supplementary Fig. S6f), suggesting a potential contribution of these signaling pathways in the embryo elongation process.

## Discussion

In this study, we comprehensively compared scRNA-seq data generated from pre-gastrulation pig, human and cynomolgus monkey conceptuses, which helped gain valuable insights into the differential transcriptional regulations during embryonic lineage transition between ungulate and primate species. Our results not only identify a developmental coordinate of pluripotency spectrum among human, cynomolgus monkey and pig embryos, and cultured human iPSCs, but also uncover potential factors and pathways that may serve as xenogeneic barriers during early development. Our study provides an invaluable informatic resource into the transcriptional divergence of early mammalian development, which in future studies may facilitate the development of effective strategies to improve the efficiency of interspecies chimera formation.

Ramos-Ibeas et al.^12^ recently reported scRNA-seq analysis of pig pre-gastrulation embryos in which several principles underlying early lineage segregation, establishment of pluripotency and X chromosome inactivation were examined. However, this study was mainly focused on pig epiblast development and lacked analysis of other tissues including TE and HYPO; moreover, pig elongation period was not included. One of the reasons why TE was not included in the analysis by Ramos-Ibeas was likely due to difficulty in effectively dissociating whole late blastocysts into single cells. To obtain single cells, late blastocysts were subjected to immunosurgery to remove the TE layer. To obtain single cells from pre-gastrulation pig embryos, Ramos-Ibeas et al. adopted a strategy that has been successfully used for rodents and primates^12^. However, the efficiency was found to be modest (average 5.5 cells/embryo). We speculated that high lipid content of pig embryos could be one of the factors associated with poor single-cell dissociation efficiency. Therefore, we developed a new method based on lipid removal and efficiently dissociated pig blastocysts into single cells (~22.6 cells/embryo) (Fig. 1b and Table 1). This method can potentially be applicable to other domestic species, e.g., cow and sheep, which also have high lipid contents in oocytes and embryos.

Mismatched developmental timing between donor PSCs and host embryos might prevent successful formation of cross-species chimera. Previous studies showed that stage-matching could overcome barriers to chimerism of gastrula-stage mouse embryos and primed human PSCs, resulting in efficient formation of interspecies chimera with proper lineage differentiation and cell dispersal^59,60^. Pearson correlation analysis revealed that hiPSCs generated using different culture conditions showed highest correlation coefficients with pig EPIs from different stages: rt2iLGoY-iPSCs more resembled pig PreL-EPIs, rNHSM-iPSCs was found more similar to pig PostE-EPIs, and primed hiPSCs clustered closer with pig PostL-EPIs (Fig. 4d; Supplementary Fig. S4c). These results suggest that rt2iLGoY-iPSCs are more compatible with EPIs from pig late blastocyst than two other types of iPSCs. It will be interesting, in future studies, to test whether rt2iLGoY-iPSCs can generate higher degree of chimerism in pig embryos.

Cell surface proteins are involved in many important biological processes, e.g., cell–cell communication and responses to external stimuli^61^. It was suggested that ligand–receptor incompatibilities and differential affinity in cell adhesion molecules might also be part of the xenogeneic barrier^4^. In our study, we found genes encoding some cell surface proteins were specifically upregulated from Pre-EPIs to Post-EPIs in pig and maintained at higher levels than those in human and monkey EPIs (Fig. 5a). Of note is that we found some receptors related to immune and inflammatory responses, and G-protein-coupled receptor activity showed different expression patterns between pig and monkey/human (Fig. 5b). We also identified differences in cell adhesion-related genes between pig and human/monkey. *IL1B* was found specifically upregulated during blastocyst-to-spheroid stage transition and maintained at high expression levels in both pig Post-EPIs and Post-TEs when compared with their human and monkey counterparts (Fig. 5b). Cell–cell communication analysis also predicated interactions IL1B_ADRB2 and IL1 receptor_IL1B were uniquely enriched in pig but not monkey PostL-EPIs, indicating IL1B may play a unique and important role in pig late epiblast development during the elongation period (Supplementary Fig. S5). In consistent with this, a previous study demonstrated that *IL1B* knockout resulted in failure of pig embryo elongation and implantation^54^. Therefore, mismatch in IL1 signaling might be one of the factors limiting human/monkey PSCs chimeric contribution to pig embryos. Similarly, tight junction-related genes also exhibited species-specific expression patterns. While *CDLN4* and *CDLN6* were highly expressed in EPIs of all three species, *CDLN9* and *CDLN10* were found specifically upregulated in pig EPIs (Fig. 5d). These cross-species differences in expression patterns of receptors and cell adhesion-related genes may compromise the survival and differentiation of donor PSCs due to their limited ability to form functional cell–cell interactions with host embryonic cells, and thereby constituting additional layers of the xenogeneic barrier. In this regard, these factors/pathways identified may provide potential targets for improving human–pig chimera formation in the future.

It was suggested that pig conceptus elongation could be mainly attributed to rapid trophoblast expansion through cellular remodeling^14^. Similarly, our GO analysis showed that genes upregulated in pig Post-TEs were enriched in terms including cell projection morphogenesis, tube development, and cell morphogenesis (Fig. 6c; Supplementary Table S7), suggesting trophoblast remodeling during this process. Interestingly, top GO terms enriched in upregulated genes in pig Post-TEs were related to neurogenesis, suggesting that pig conceptus elongation shares similar regulators and/or pathways with neuron differentiation and axon growth (Fig. 6c; Supplementary Table S7). In addition, we found cell cycle- and DNA replication-related genes were significantly upregulated from Pre-TEs to PostE- and PostL-TEs, which indicates rapid cell proliferation (Fig. 6d). These results suggest that pig conceptus elongation involves both dramatic morphological changes and high cell proliferation in trophoblasts. In addition to TE, we found cell cycle- and DNA replication-related genes were also upregulated in pig but not in human/monkey Post-EPIs (Supplementary Fig. S6c). There results suggest cell proliferation rates are different between human/monkey and pig conceptus during this period, which likely also contributes to the xenogeneic barrier between human and pig.

In conclusion, we provide a systematic comparative analysis of early development among pig, monkey, and human. Our analyses identify species-specific differences during several pre-gastrulation developmental stages. Future studies are warranted to functionally validate these differences and study the roles they play in xenogeneic barriers between primates and pigs. These results offer new insights into evolutionary conserved and divergent processes during mammalian development and maybe helpful for developing effective strategies to overcome low human–pig chimerism, and thereby enabling the generation of functional human organs in pigs in the future.

## Materials and methods

### Wuzhishan pig (WZSP)

WZSP is a mini pig distributed in Hainan province of China and the sequencing of its genome was completed in 2012^62^. Embryos used in this paper were derived from WZSPs, which were from BGI Ark Biotechnology Co., LTD (BAB). All animal experimental procedures were performed with the approval of the Life Ethics and Biological Safety Review Committee of BGI-Research.

### Embryo collection and in vitro culture

The estrus signs of donor pig were checked and recorded every day. The donors were artificially inseminated one time at the beginning of estrus, and second time 12 h later. One-cell stage embryos were collected from a living donor and then cultured in the incubator until being dispersed into single cells after 5 days or 7 days. Briefly, 1 day after the donor was inseminated, donors were opened abdomen surgically, putative embryos on one-cell stage were collected at ampulla of oviduct by injecting phosphate buffer solution (PBS) (Invitrogen) supplemented with 3% fetal bovine serum (Hyclone) to flush the embryos from uterine horn to oviduct. The recovered embryos were transported to laboratory in 4 h and cultured in PZM-3 (Sigma) supplemented with 4 mg/mL bovine serum albumin (BSA) (Sigma) at 38.5 °C in an atmosphere of 5% CO_2_, 5% O_2_. Embryos on the stage of 10 days or 12 days were recovered by sacrificing donor pig. Briefly, the sows were killed at 10 or 12 days after artificial insemination. the uterine tracts were transported to laboratory in 4 h at 38.5 °C in PBS. Uterine horns attached to the mesometrium were released with scissors and the cervix was clamped before this end was cut. Then PBS supplemented with 3% fetal bovine serum was injected into the end of the uterine horns to flush the uterine content with a gentle massage. After that about 150 mL flush buffer was injected with a gentle massage, the clamp on the cervix was taken off, the flush buffer was collected into a sterile vessel. Embryos in the flush were checked and picked up under a microscope (Olympus).

### Individual cell isolation and single-cell cDNA preparation

Day 5 and day 7 embryos were washed three times in PBS to remove culture medium. Those embryos used to dissect inner cell mass (ICM) were digested with 3 mg/mL pronase (Sigma-Aldrich) to remove Zona, and then washed three times in T20 (T is HEPES buffered TCM-199 medium; the number is the percentage (vol/vol) of calf serum supplementation) to stop digestion. The ICM was dissected with a little fine shape blade under a microscope. After that, intact embryos and dissected inner cell mass were further treated with one of the following three methods: (1) digesting only with enzymes including tripsin (0.05% and 0.25%) (Sigma), pronase (Sigma), hyaluronidase (Sigma), IV collagenase (Sigma) for 10–60 min complying with repeated pipetting; (2) placed in EDTA (Sigma) for 5 min before digested with enzyme solution for 10–15 min with repeated pipetting; (3) placed in EDTA for 5 min after washed in PBS for three times, and then transferred to 1.5 mL tubes filled with accutase solution containing 7.5 μg/mL cytochalasin B, centrifuged for 30 min at 1200× *g*. After that, single cells were recovered and the remains were further digested in accutase solution without cytochalasin B with repeated pipetting at 38.5 °C.

Day 10 or day 12 embryos were transferred to a dish filled with PBS, and then the epiblasts were gently dissected from the other parts of the embryo with fine forceps and needles. As porcine day 12 embryos were on the elongation stage, the structure was complicated, it took more time and patience to perform dissecting. The epiblasts (may attach to a few cells of other tissue) were washed three times in PBS supplemented with 0.03% BSA before digested with 0.05% trypsin at 38.5 °C for 10 min, then epiblasts were repeatedly pipetted. The single cells were collected with a finely pulled glass tip and quickly transferred into lysis buffer following reverse transcription-polymerase chain reaction, sequence-specific reverse transcription, and pre-amplification polymerase chain reaction. Smart-seq2 method was applied as described previously^63^.

### Assessment of apoptosis and cell death

Propidium iodide (PI, 4 μmol/L) and calcein acetoxymethylester (Calcein-AM, 2 μmol/L) (Sigma-Aldrich, Inc.) were used as fluorescent markers to assess cell state. Cell viability was determined by charalcein-AM, for it can be transported through the cellular membrane of live cells, while PI, a membrane-impermeant fluorescent dye, which was commonly excluded from live cells, was used to determine dead cells. Two dyes were added to the solution containing single cells simultaneously. The cellular state was examined under a fluorescence microscope (Olympus). The results of fluorescence microscope imaging were shown in Fig. 1c. The live cells were shown in green (Calein-AM), and the dead cells were shown in red (PI). The percentage of viable cells was approximately over 80% after cell experiments.

### Lineage identification of cells in Pig

scRNA-seq reads were mapped to the WZSP reference genome (Minipig_v3) using HISAT2^64^ (version 2.0.4) with parameters “-k 1 --min-intronlen 20 --max-intronlen 120000”. Then the expression levels of each gene were calculated by the fragments per kilobase of exons per million fragments mapped (FPKM) using StringTie^65^ with parameters “-t -C -e -B -A” based on the result of HISAT2. In all, 16,272 genes were detected in total, and out of them 10,794 homologous genes between pig and monkey/human were used to perform cross-species comparative analysis (Supplementary Table S10). The expression matrix of Homo sapiens (human) and Macaca fascicularis (monkey) were downloaded from GSE109555^19^ and GSE74767^18^, respectively. The raw data of human induced pluripotent stem cells (hiPSCs) were downloaded from SRP115256^34^ and the gene expression levels were calculated in the same way (GRCh38.p12 was used as human reference genome). Unsupervised hierarchical clustering (UHC) was performed with genes whose FPKM meets the standard: log_2_(FPKM + 1) > 4 in at least one cell^34^. The number of possible cell types from different lineages at different developmental stages were estimated based on previous literature^44^, and known lineage marker genes were used to define different clusters in our bioinformatics results^25–28^. The distance used was Euclidian, and the cluster method was Ward’s method (ward.D2). Pseudotime analysis was performed by using the Monocle2 R package^66^ in each pig lineage (EPI, TE, and HYPO).

### Cross-species comparative analysis

A twofold variance in FPKM and an adjusted *P*-value < 0.05 were used as cutoffs to define DEGs in EPI of each species. A 2.8-fold variance in FPKM and an adjusted *P*-value < 0.05 were used as cutoffs to define DEGs in TE of each species. The adjusted *P*-value was calculated using the Wilcoxon test. After identifying DEGs independently in different transitions for each species, we determined the species-specific differentially expressed genes to perform the downstream analysis. GO and KEGG analyses were performed using the R software (clusterProfiler), and since the annotation of monkey was relatively incomplete, the human annotation was used for functional analysis of monkey. PCA and heatmap analysis were performed using R software. The final cross-species expression matrix was calculated by cross-species gene expression analysis as reported previously^67^. Pearson’s correlation analysis was carried out with the final cross-species expression matrix using R software. CellPhoneDB^45^ was used to predict the interaction of receptors and ligands in different cell types in each species. The annotation of surface markers was downloaded from Cell Surface Protein Atlas (CSPA) (https://wlab.ethz.ch/cspa/)^68^ to help find the species-specific surface proteins.

## Supplementary information

Supplementary materials

Supplementary Table S1

Supplementary Table S2

Supplementary Table S3

Supplementary Table S4

Supplementary Table S5

Supplementary Table S6

Supplementary Table S7

Supplementary Table S8

Supplementary Table S9

Supplementary Table S10

## Data Availability

The scRNA-seq data of pig embryos that support the findings of this study have been deposited in the CNSA (https://db.cngb.org/cnsa/) of CNGBdb with accession code CNP0000872.

## Supplementary information

**Supplementary Information** accompanies the paper at (10.1038/s41421-020-00238-x).
