# Author Correction: Cross-species single-cell transcriptomic analysis reveals pre-gastrulation developmental differences among pigs, monkeys, and humans

**DOI:** 10.1038/s41421-021-00251-8

**Published:** 2021-03-12

**Authors:** Tianbin Liu, Jie Li, Leqian Yu, Hai-Xi Sun, Jing Li, Guoyi Dong, Yingying Hu, Yong Li, Yue Shen, Jun Wu, Ying Gu

**Affiliations:** 1 BGI Education Center, University of Chinese Academy of Sciences, Shenzhen, Guangdong 518083 China; 2 BGI-Shenzhen, Shenzhen, Guangdong 518083 China; 3 Guangdong Provincial Key Laboratory of Genome Read and Write, BGIShenzhen, Shenzhen, Guangdong 518120 China; 4 Department of Molecular Biology, University of Texas Southwestern Medical Center, Dallas, TX 75390 USA; 5 Hamon Center for Regenerative Science and Medicine, University of Texas Southwestern Medical Center, Dallas, TX 75390 USA; 6 BGI Institute of Applied Agriculture, BGI-Shenzhen, Shenzhen, Guangdong 518120 China; 7 Institute for Stem cell and Regeneration, Chinese Academy of Sciences, Beijing, 100101 China

**Keywords:** Pluripotency, Cell growth, Cell signalling

Correction to: *Cell Discovery* (2021) 7:8

10.1038/s41421-020-00238-x Published online 02 February 2021

In the original publication of this article^1^, the description of data source and reference for the identification of epithelial cell lineages of pigs were missing and should be added in Materials and methods section as follows.

**Lineage identification of cells in Pig**

The expression matrix of the endometrial epithelium, including the pregnant glandular epithelium (GSM2946041-44), the pregnant luminal epithelium (GSM2946045-48), the control glandular epithelium (GSM2946030-33), and the control luminal epithelium (GSM2946034-37), was downloaded from GSE109539^2^.

The newly added reference^2^ should be cited in the main text as shown below.

“To investigate potential roles of the endometrial epithelium may play in pig embryo elongation, we used CellPhoneDB^45^ to predict the ligand–receptor interactions between pregnant womb epithelium (luminal and glandular, P_LE and P_GE)^2^ and PostL-EPIs or PostL-TEs during the elongation stages”.

In addition, in the last Venn diagram of Fig. 5c, the labels of 52 and 169 were misplaced and should be interchanged as shown below.

**Fig. 5:**
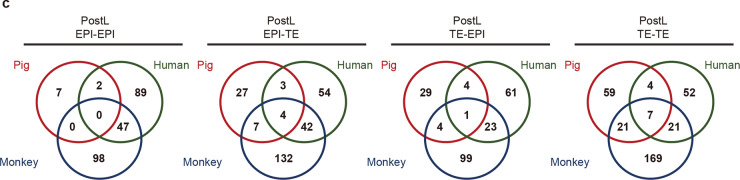
Cross-species comparison of membrane-related genes among pig, monkey and human EPIs. **c** Overlaps of the interaction relationship between PostL-EPIs and PostL-TEs in each species. A total of 59, 52, and 169 pairs were predicted to be unique between Post-TEs (TE–TE) in pig, human, and monkey, respectively.

## References

[1] Liu T (2021). Cross-species single-cell transcriptomic analysis reveals pre-gastrulation developmental differences among pigs, monkeys, and humans. Cell Discov..

[2] Zeng S, Bick J, Ulbrich SE, Bauersachs S (2018). Cell type-specific analysis of transcriptome changes in the porcine endometrium on Day 12 of pregnancy. BMC Genomics.

