# Supplementary material for: Cross-species single-cell transcriptomic analysis reveals pre-gastrulation developmental differences among pigs, monkeys, and humans
Source: Cell Discov. 2021 Feb 2;7:8. doi: 10.1038/s41421-020-00238-x (PMC7854681; doi:10.1038/s41421-020-00238-x)
Supplement: Supplementary file 1 — Supplementary materials [file 41421_2020_238_MOESM1_ESM.pdf]

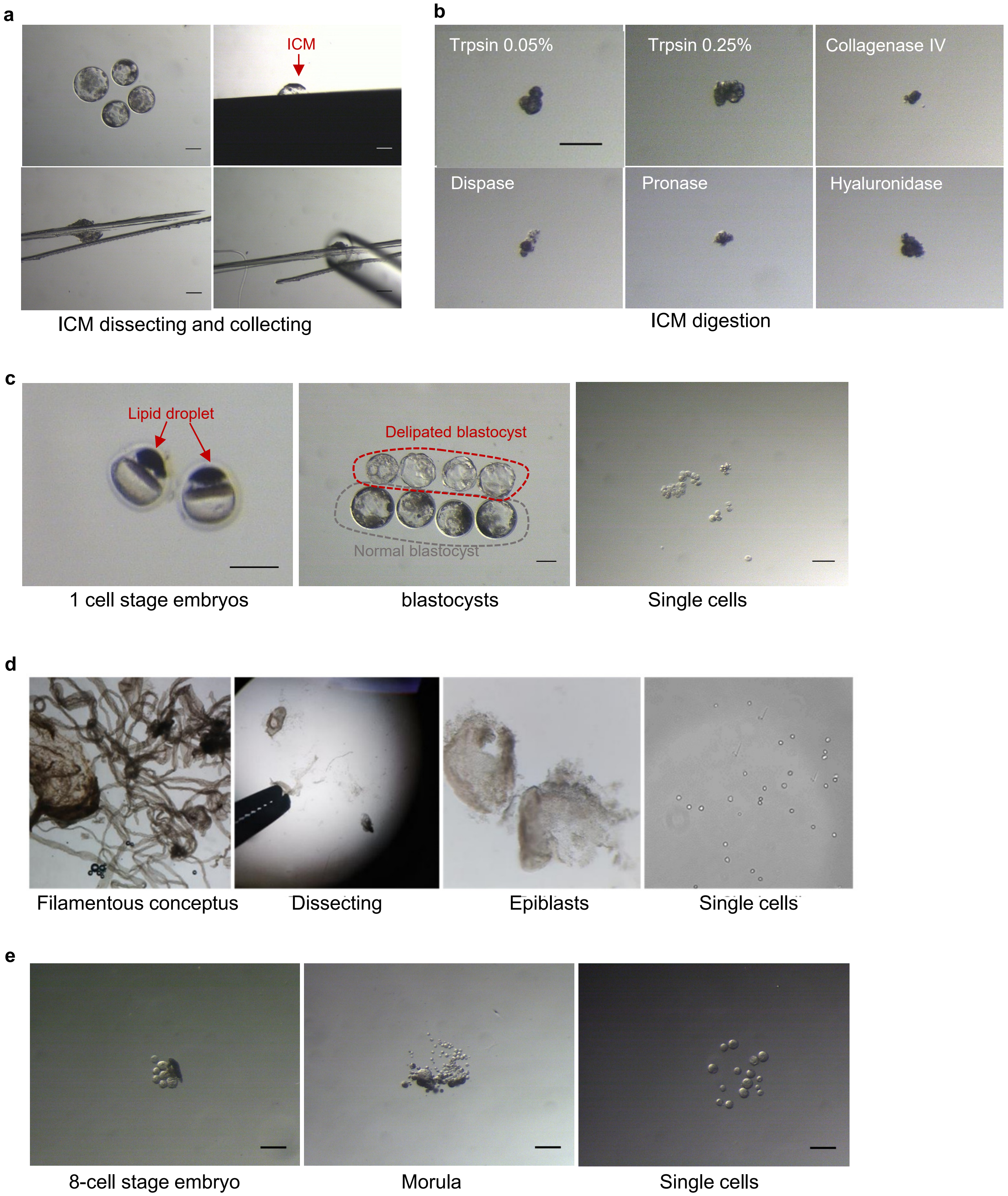
a
ICM
ICM dissecting and collecting
b
Trpsin 0.05%
Trpsin 0.25%
Collagenase IV
Dispase
Pronase
Hyaluronidase
ICM digestion
c
Lipid droplet
Delipated blastocyst
Normal blastocyst
1 cell stage embryos
blastocysts
Single cells
d
Filamentous conceptus
Dissecting
Epiblasts
Single cells
e
8-cell stage embryo
Morula
Single cells

Fig. S1 Optimization of pig embryo single cell dissociation protocol. a Represent images showing isolation of pig ICM with microblade. Bar represents 100 μm. **b** Digesting ICM with different types of enzymes. Bar represents 100 μm. **c** Dissociating blastocyst developing from 1-cell stage embryo delipated (lipid removal) before in vitro culture. Bar represents 100 μm. **d** Represent images showing dissecting and dissociating day 12 pig conceptus. e Represent images showing dissociation of pig embryos at earlier stages (8-cell and morulae). Bar represents 100 μm.

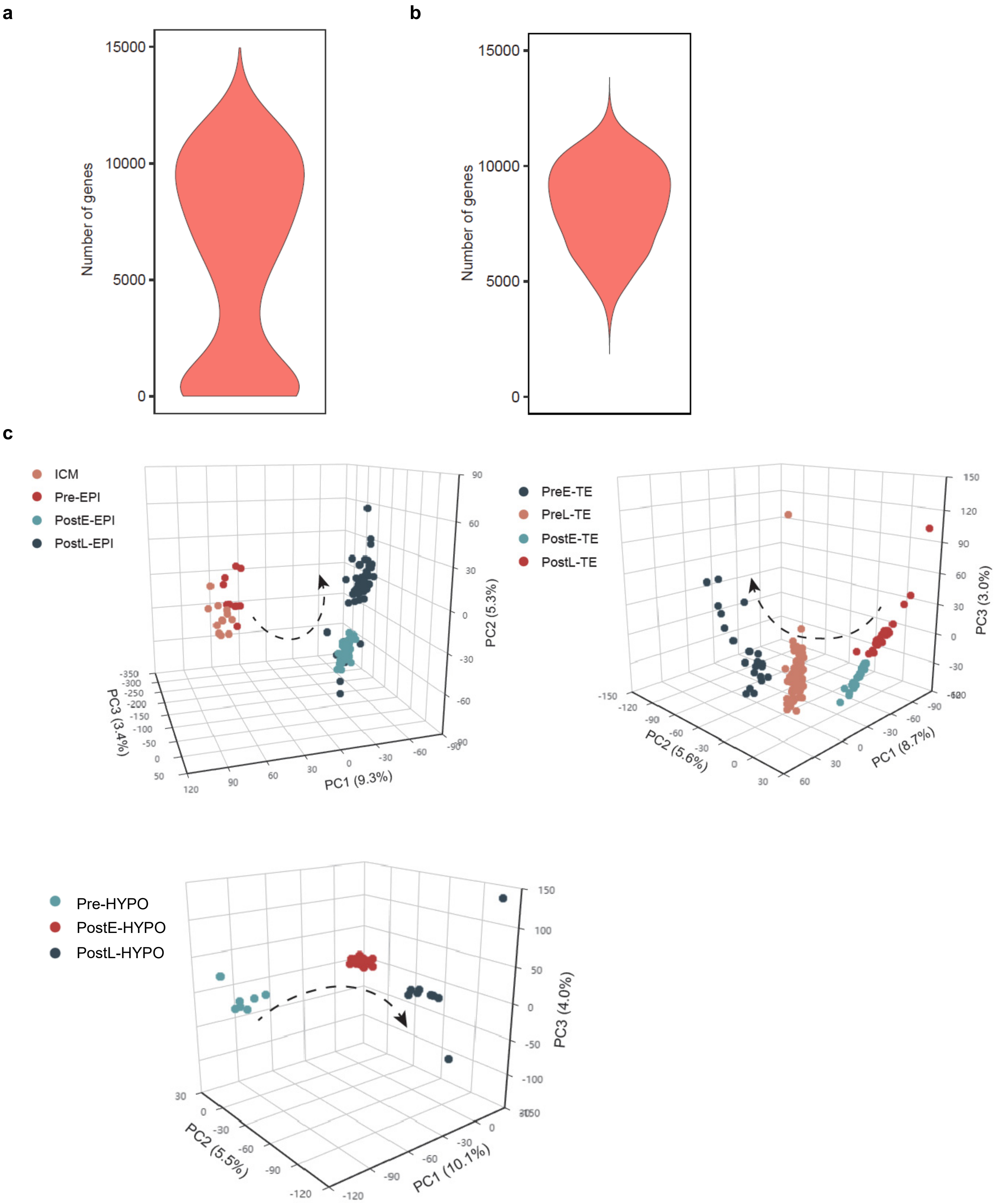
a
b
c
Number of genes
15000
10000
5000
0
ICM
Pre-EPI
PostE-EPI
PostL-EPI
PC1 (9.3%)
PC2 (5.3%)
PC3 (3.4%)
PreE-TE
PreL-TE
PostE-TE
PostL-TE
PC1 (8.7%)
PC2 (5.6%)
PC3 (3.0%)
Pre-HYPO
PostE-HYPO
PostL-HYPO
PC1 (10.1%)
PC2 (5.5%)
PC3 (4.0%)

Fig. S2 Cell quality check and principal component analysis (PCA). a Violin plots of gene numbers detected in all cells. **b** Violin plots of gene numbers in those cells used in the downstream analysis. **c** PCA of of the pig EPI, HYPO and TE, respectively. Stage information was indicated as follows: Pre- (Day 5 or/and Day 7), PreE- (Day 5); PreL- (Day 7), Post- (Day 10 and Day 12), PostE- (Day 10); PostL- (Day12).

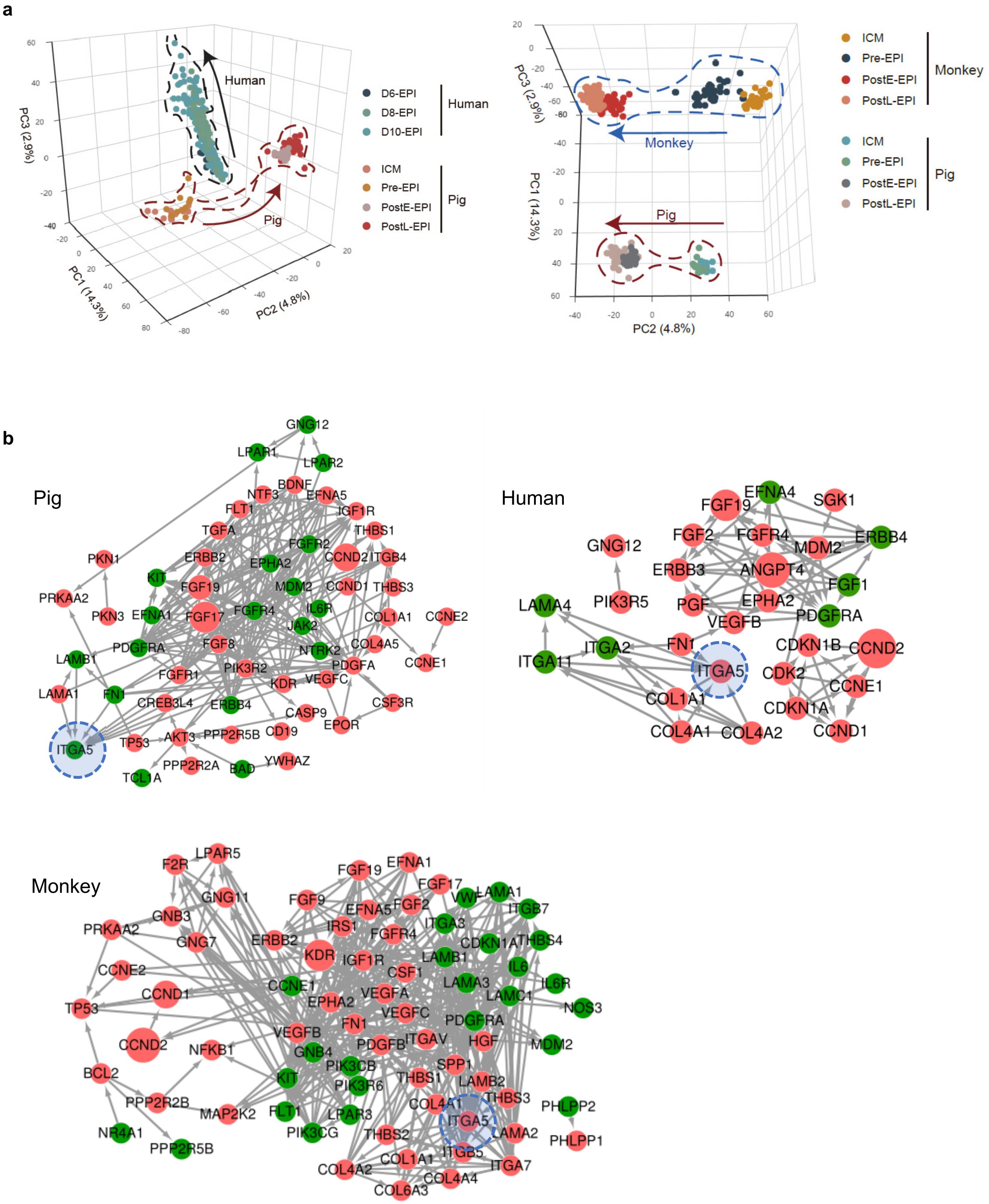

a
Human
Pig
PC3 (2.9%)
PC1 (14.3%)
PC2 (4.8%)
D6-EPI
D8-EPI
D10-EPI
Human
ICM
Pre-EPI
PostE-EPI
PostL-EPI
Pig
Monkey
Pig
ICM
Pre-EPI
PostE-EPI
PostL-EPI
Monkey
ICM
Pre-EPI
PostE-EPI
PostL-EPI
Pig
b
Pig
Human
Monkey
ITGA5

Fig. S3 PCA analysis of EPI development and comparison of PPI network of "PI3K-Akt signaling pathway between pig, monkey and human. a PCA of pig and human orthologous genes expressed in EPIs. PCA of pig and monkey orthologous genes expressed in EPIs. **b** PPI network of "PI3K-Akt signaling pathway" in each species. Stage information was indicated as follows: Pre- (Day 5 or/and Day 7), PreE- (Day 5); PreL- (Day 7), Post- (Day 10 and Day 12), PostE- (Day 10), PostL- (Day12).

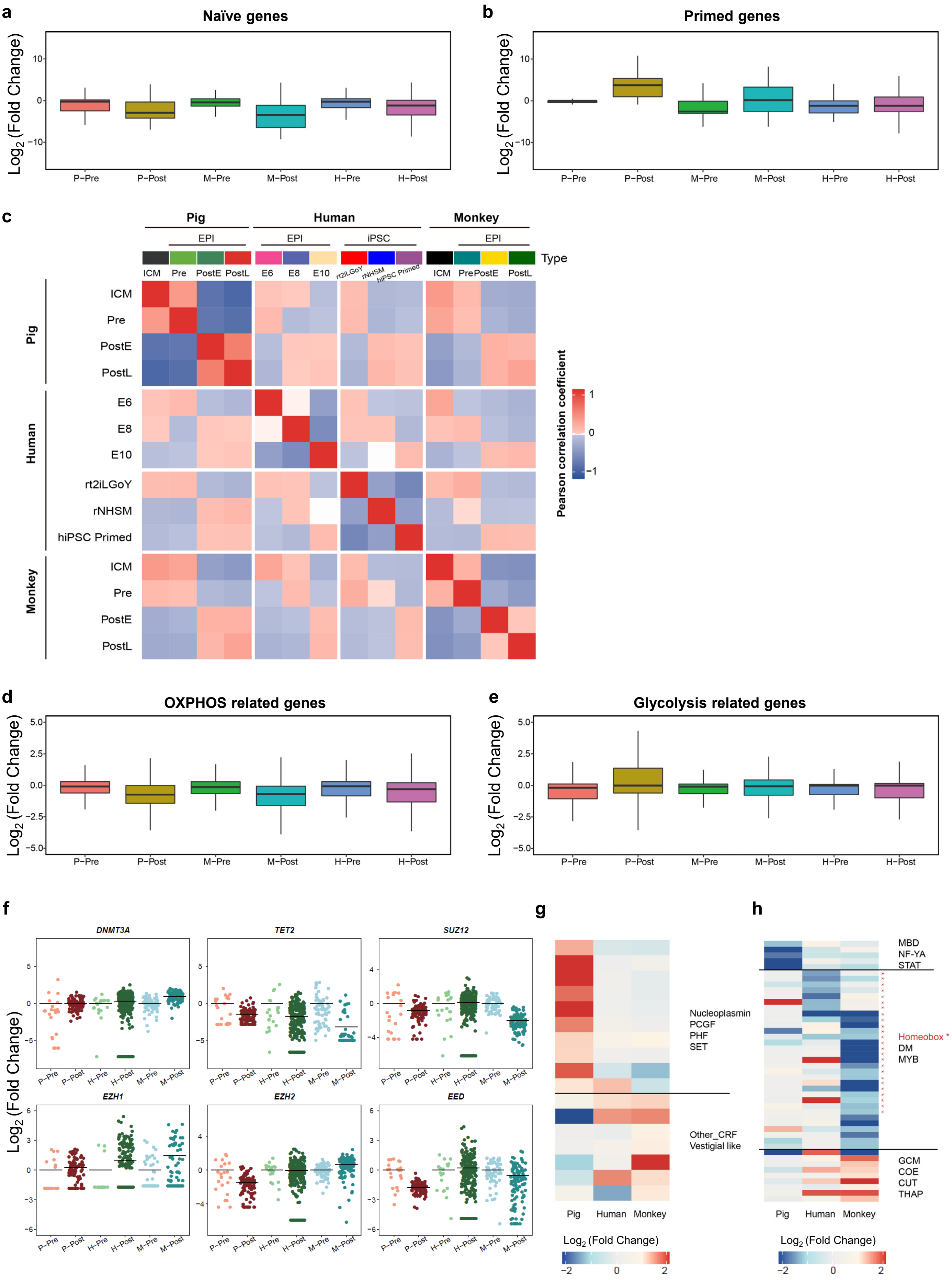
a
Naïve genes
Log2 (Fold Change)
10
0
−10
P−Pre
P−Post
M−Pre
M−Post
H−Pre
H−Post
b
Primed genes
Log2 (Fold Change)
c
Pig
Human
Monkey
EPI
iPSC
Type
ICM
Pre
PostE
PostL
E6
E8
E10
rt2iLGoY
rNHSM
hiPSC Primed
Pearson correlation coefficient
1
0
−1
d
OXPHOS related genes
Log2 (Fold Change)
5.0
2.5
0.0
−2.5
−5.0
e
Glycolysis related genes
f
DNMT3A
TET2
SUZ12
EZH1
EZH2
EED
Log2 (Fold Change)
g
Nucleoplasmin
PCGF
PHF
SET
Other_CRF
Vestigial like
Pig
Human
Monkey
Log2 (Fold Change)
−2
0
2
h
MBD
NF-YA
STAT
Homeobox *
DM
MYB
GCM
COE
CUT
THAP

Fig. S4 Pluripotency, OXPHOS, glycolysis and transcriptional genes expression regulations. a Box plot of naïve pluripotency related genes in each species. **b** Box plot of primed pluripotency related genes in each species. P-Pre, Pig ICM and Pre- EPIs; P-Post, Pig PostE- and PostL- EPIs; H-Pre, Human D6-EPIs; H-Post, Human D8- and D10- EPIs; M-Pre, Monkey ICM and Pre-EPIs; M-Post, Monkey PostE- and PostL- EPIs. **c** Heatmap of the correlation coefficients among EPIs and hiPSCs. **d** Box plot of OXPHOS related genes in each species. **e** Box plot of glycolysis related genes in each species. **f** Scatter-plot of species-specific methylation related genes. **g** Heatmap of differential analysis of species-specific cofactor related genes in Pre- and Post- EPIs of each species. **h** Heatmap of differential analysis of species-specific TF related genes in Pre- and Post- EPIs of each species. Stage information was indicated as follows: Pre- (Day 5 or/and Day 7), PreE- (Day 5); PreL- (Day 7), Post- (Day 10 and Day 12), PostE- (Day 10), PostL- (Day12). The horizontal lines in **f** indicate the mean values. To avoid the influence caused by the "0" value, a new matrix was generated by using FPKM+1 and the $\log_2$-transformed fold change was calculated.

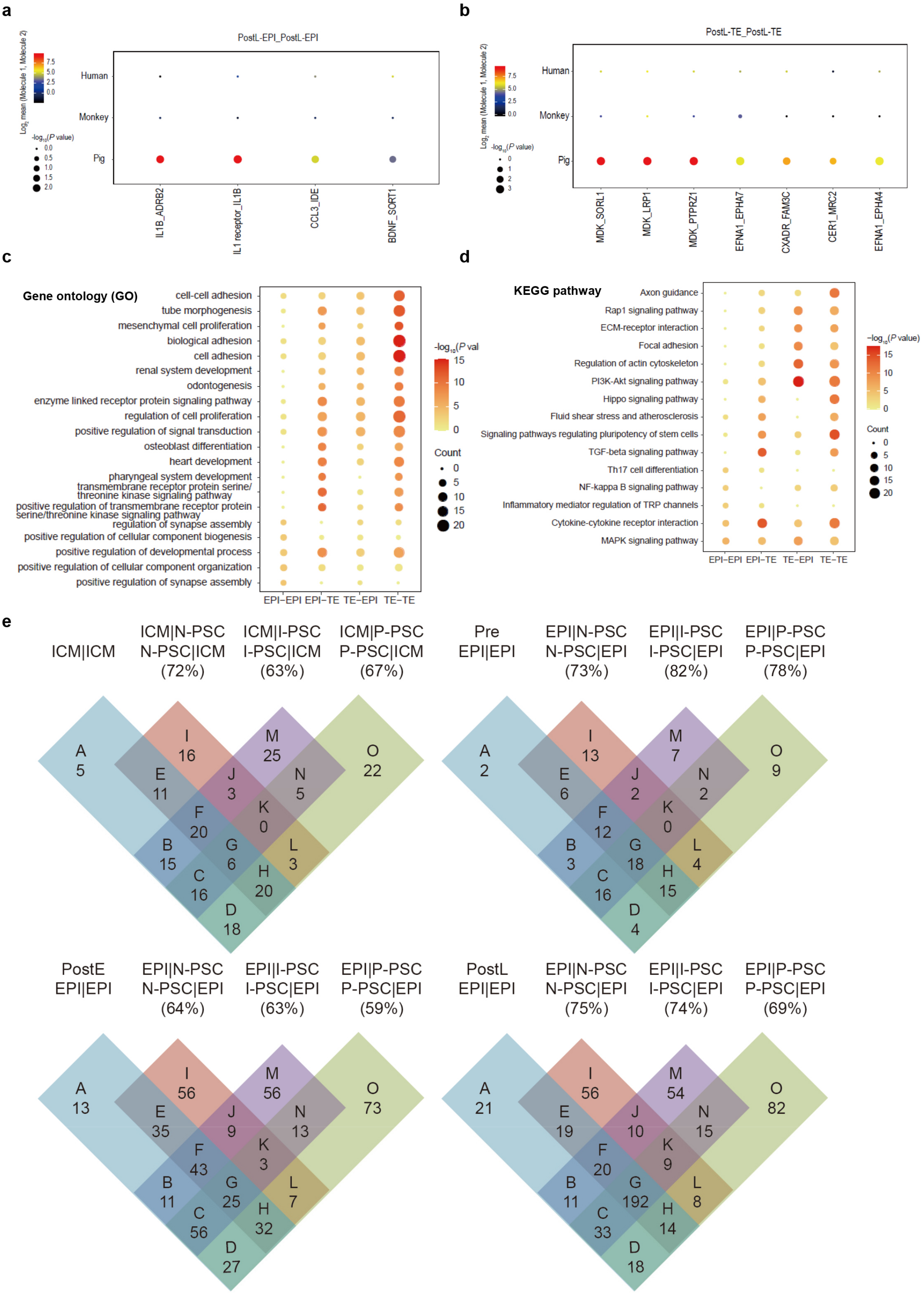
a
PostL-EPI_PostL-EPI
Human
Monkey
Pig
IL1B_ADRB2
IL1 receptor_IL1B
CCL3_IDE
BDNF_SORT1
b
PostL-TE_PostL-TE
MDK_SORL1
MDK_LRP1
MDK_PTPRZ1
EFNA1_EPHA7
CXADR_FAM3C
CER1_MRC2
EFNA1_EPHA4
c
Gene ontology (GO)
cell-cell adhesion
tube morphogenesis
mesenchymal cell proliferation
biological adhesion
cell adhesion
renal system development
odontogenesis
enzyme linked receptor protein signaling pathway
regulation of cell proliferation
positive regulation of signal transduction
osteoblast differentiation
heart development
pharyngeal system development
transmembrane receptor protein serine/threonine kinase signaling pathway
positive regulation of transmembrane receptor protein serine/threonine kinase signaling pathway
regulation of synapse assembly
positive regulation of cellular component biogenesis
positive regulation of developmental process
positive regulation of cellular component organization
positive regulation of synapse assembly
EPI−EPI EPI−TE TE−EPI TE−TE
Count
d
KEGG pathway
Axon guidance
Rap1 signaling pathway
ECM-receptor interaction
Focal adhesion
Regulation of actin cytoskeleton
PI3K-Akt signaling pathway
Hippo signaling pathway
Fluid shear stress and atherosclerosis
Signaling pathways regulating pluripotency of stem cells
TGF-beta signaling pathway
Th17 cell differentiation
NF-kappa B signaling pathway
Inflammatory mediator regulation of TRP channels
Cytokine-cytokine receptor interaction
MAPK signaling pathway
EPI−EPI EPI−TE TE−EPI TE−TE
Count
e
ICM|ICM
ICM|N-PSC N-PSC|ICM (72%)
ICM|I-PSC I-PSC|ICM (63%)
ICM|P-PSC P-PSC|ICM (67%)
A 5, I 16, M 25, O 22, E 11, J 3, N 5, F 20, K 0, B 15, G 6, L 3, C 16, H 20, D 18
Pre EPI|EPI
EPI|N-PSC N-PSC|EPI (73%)
EPI|I-PSC I-PSC|EPI (82%)
EPI|P-PSC P-PSC|EPI (78%)
A 2, I 13, M 7, O 9, E 6, J 2, N 2, F 12, K 0, B 3, G 18, L 4, C 16, H 15, D 4
PostE EPI|EPI
EPI|N-PSC N-PSC|EPI (64%)
EPI|I-PSC I-PSC|EPI (63%)
EPI|P-PSC P-PSC|EPI (59%)
A 13, I 56, M 56, O 73, E 35, J 9, N 13, F 43, K 3, B 11, G 25, L 7, C 56, H 32, D 27
PostL EPI|EPI
EPI|N-PSC N-PSC|EPI (75%)
EPI|I-PSC I-PSC|EPI (74%)
EPI|P-PSC P-PSC|EPI (69%)
A 21, I 56, M 54, O 82, E 19, J 10, N 15, F 20, K 9, B 11, G 192, L 8, C 33, H 14, D 18

Fig. S5 Analysis of cell interaction in/between EPI and TE. a Bubble chart of selected ligand–receptor interactions from PostL-EPI_PostL-EPI. **b** Bubble chart of selected ligand–receptor interactions from PostL-TE_PostL-TE. **c** GO term analyses of pig-specific interaction relationships between PostL-EPIs and PostL-TEs. **d** Pathway enrichment analyses of pig-specific interaction relationships between PostL-EPIs and PostL-TEs. **e** Overlaps of the cell-cell interactions between EPIs, Naive PSCs (N-PSC), intermediate PCSs (I-PSC) and Primed PSCs (P-PSC) in each stage, percent of overlapped interactions between EPI and PSCs were showed in each group. Stage information was indicated as follows: Pre- (Day 5 or/and Day 7), PreE- (Day 5); PreL- (Day 7), Post- (Day 10 and Day 12), PostE- (Day 10), PostL- (Day12).

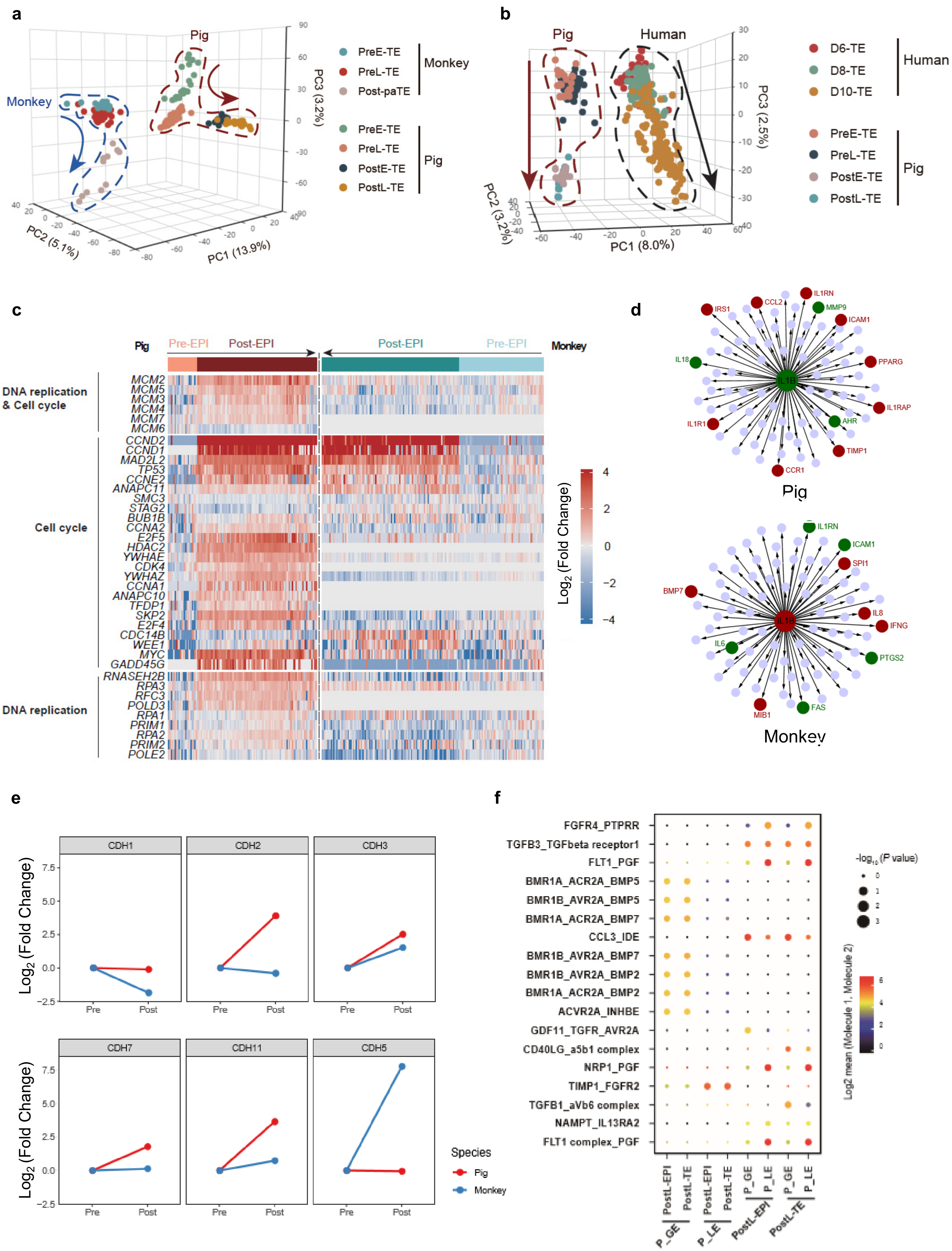
a
Monkey
Pig
PreE-TE
PreL-TE
Post-paTE
Monkey
PreE-TE
PreL-TE
PostE-TE
PostL-TE
Pig
PC3 (3.2%)
PC2 (5.1%)
PC1 (13.9%)
b
Pig
Human
D6-TE
D8-TE
D10-TE
Human
PreE-TE
PreL-TE
PostE-TE
PostL-TE
Pig
PC3 (2.5%)
PC2 (3.2%)
PC1 (8.0%)
c
Pig
Pre-EPI
Post-EPI
Post-EPI
Pre-EPI
Monkey
DNA replication & Cell cycle
MCM2
MCM5
MCM3
MCM4
MCM7
MCM6
Cell cycle
CCND2
CCND1
MAD2L2
TP53
CCNE2
ANAPC11
SMC3
STAG2
BUB1B
CCNA2
E2F5
HDAC2
YWHAE
CDK4
YWHAZ
CCNA1
ANAPC10
TFDP1
SKP2
E2F4
CDC14B
WEE1
MYC
GADD45G
DNA replication
RNASEH2B
RPA3
RFC3
POLD3
RPA1
PRIM1
RPA2
PRIM2
POLE2
Log2 (Fold Change)
4
2
0
−2
−4
d
IRS1
CCL2
IL1RN
MMP9
ICAM1
IL18
PPARG
IL1B
IL1RAP
AHR
IL1R1
TIMP1
CCR1
Pig
IL1RN
ICAM1
SPI1
BMP7
IL8
IFNG
IL1B
IL6
PTGS2
MIB1
FAS
Monkey
e
CDH1
CDH2
CDH3
CDH7
CDH11
CDH5
Log2 (Fold Change)
Pre
Post
Species
Pig
Monkey
f
FGFR4_PTPRR
TGFB3_TGFbeta receptor1
FLT1_PGF
BMR1A_ACR2A_BMP5
BMR1B_AVR2A_BMP5
BMR1A_ACR2A_BMP7
CCL3_IDE
BMR1B_AVR2A_BMP7
BMR1B_AVR2A_BMP2
BMR1A_ACR2A_BMP2
ACVR2A_INHBE
GDF11_TGFR_AVR2A
CD40LG_a5b1 complex
NRP1_PGF
TIMP1_FGFR2
TGFB1_aVb6 complex
NAMPT_IL13RA2
FLT1 complex_PGF
PostL-EPI
PostL-TE
PostL-EPI
PostL-TE
P_GE
P_LE
P_GE
P_LE
P_GE
P_LE
PostL-EPI
PostL-TE
-log10 (P value)
Log2 mean (Molecule 1, Molecule 2)

Fig. S6 Genes regulation of pig EPI and TE during the Elongation related genes. **a** PCA of pig and monkey orthologous genes expressed in TEs. **b** PCA of pig and human orthologous genes expressed in TEs. **c** Heatmap of difference folds of cell cycle and DNA replication related genes in Pre- and Post- EPIs of pig and monkey. **d** The network of IL1B and its target genes in pig and monkey. Using Pre-TEs as a control, down- and up-regulated genes in Post-TEs are shown in blue and red. **e** Line chart showing the fold change of CDH families in Pre- and Post- TEs of pig and monkey. **f** Bubble chart of selected ligand–receptor interactions in the pregnant womb. Stage information was indicated as follows: Pre- (Day 5 or/and Day 7), PreE- (Day 5); PreL- (Day 7), Post- (Day 10 and Day 12), PostE- (Day 10), PostL- (Day12).
